# PCSK9 promotes progression of anaplastic thyroid cancer through E-cadherin endocytosis

**DOI:** 10.1038/s41419-025-07690-1

**Published:** 2025-05-06

**Authors:** Yu Zhang, Wei Su, Xiaoyu Ji, Zhou Yang, Qing Guan, Yuanxin Pang, Linkun Zhong, Yu Wang, Jun Xiang

**Affiliations:** 1 Department of Head and Neck Surgery, Fudan University Shanghai Cancer Center, Shanghai, 200032 China; 2 Department of Oncology, Shanghai Medical College, Fudan University, Shanghai, 200032 China; 3 Department of Medical Oncology, Fudan University Shanghai Cancer Center, Shanghai, China; 4 Department of Oncology, Huashan Hospital Fudan University, Shanghai, China; 5 Department of Endocrinology, Suzhou Ninth People’s Hospital Affiliated to Soochow University, Suzhou, China; 6 Department of General Surgery, Zhongshan City People’s Hospital, Zhongshan, Guangdong Province China

**Keywords:** Biochemistry, Cancer therapy

## Abstract

Although anaplastic thyroid cancer (ATC) constitutes only 1–2% of all thyroid malignancies, it is associated with an exceptionally high mortality rate, accounting for 14–39% of thyroid cancer-related deaths. In this study, we identified the critical role of Proprotein Convertase Subtilisin/Kexin Type 9 (PCSK9) in ATC progression. Proteomic analysis revealed E-cadherin as a key mediator of PCSK9-driven malignancy in ATC. Mechanistically, PCSK9 promotes the degradation of E-cadherin through the lysosomal pathway. Furthermore, the loss of the p53 function, particularly the R248Q mutation, de-repressed *PCSK9* expression at the transcriptional level. Notably, the PCSK9 inhibitor PF-846 considerably suppressed ATC proliferation and metastasis in both in vitro and in vivo models. In conclusion, PCSK9 enhances ATC malignancy by regulating E-cadherin degradation via the lysosomal pathway, underscoring its potential as a promising therapeutic target.

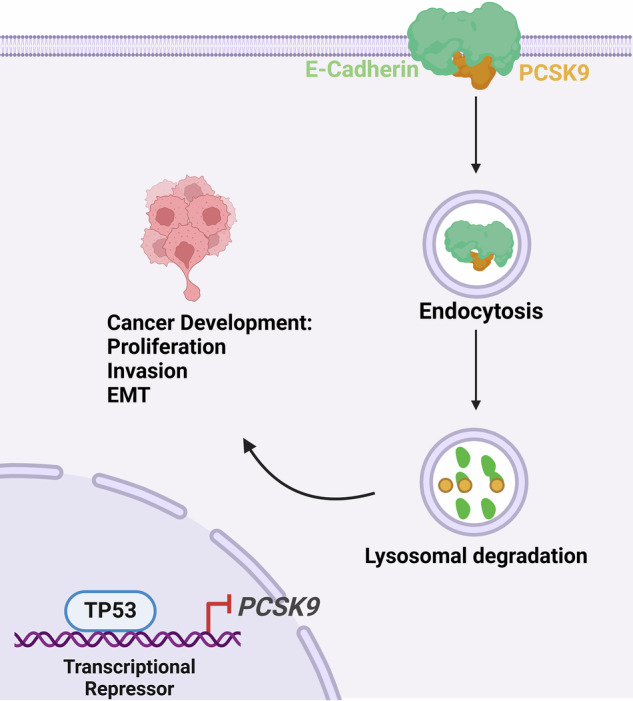

## Introduction

Thyroid cancer (TC) is one of the most common malignancies of the endocrine system. Over the past decade, the global incidence of TC has risen by approximately 20% [1, 2]. TC includes several pathological types: papillary TC (PTC), follicular TC (FTC), medullary TC, and anaplastic TC (ATC). Differentiated TC (DTC), which primarily includes PTC and FTC, accounts for approximately 95% of all cases. Standard treatments—surgery, endocrine therapy, and radioactive iodine therapy—achieve favorable outcomes, with 10-year survival rates exceeding 90% [3].

However, ATC represents a distinct and formidable challenge. Although ATC accounts for only 1–2% of all thyroid malignancies, it is responsible for 14–39% of TC-related deaths [4, 5]. The median survival time is less than 6 months from diagnosis, and some patients succumb rapidly because of aggressive tumor growth invading critical structures such as the trachea and esophagus. Achieving R0/R1 resection is difficult in most cases because of frequent invasion or encasement of adjacent structures, including the internal jugular vein, carotid artery, and larynx [6].

Although targeted therapies such as BRAF inhibitors show potential, especially in patients with ATC with BRAF mutations, challenges remain. For example, the combination of dabrafenib and the MEK1/2 inhibitor trametinib has resulted in significant tumor regression in some cases. Additionally, immunotherapy has opened new treatment avenues [7, 8]. Yet, treatment resistance and management of patients with wild type BRAF ATC continue to pose significant hurdles [9]. These challenges highlight the urgent need to develop more specific and effective therapies for ATC.

Proprotein convertase subtilisin/kexin type 9 (PCSK9), a member of the proteinase K family of subtilases, is primarily synthesized in the liver and small intestine [10]. It undergoes autocatalytic cleavage in the endoplasmic reticulum, a process critical for its secretion and functional activity [11, 12]. PCSK9 forms a complex with low-density lipoprotein (LDL) receptor (LDLR) in acidic endosomes, promoting LDLR degradation and thereby influencing LDL metabolism. As such, PCSK9 has emerged as a therapeutic target for hypercholesterolemia and cardiovascular disease prevention [13, 14]. Emerging evidence indicates that PCSK9 exhibits aberrant expression in various cancers and is closely associated with patient prognosis [15, 16]. Furthermore, recent studies suggest that PCSK9 may serve as a target for cancer immunotherapy [17]. However, its potential role in ATC remains unexplored.

Targeted therapies continue to hold promise in both preoperative and postoperative settings for ATC [18, 19]. Nonetheless, many patients face limited efficacy or resistance to such treatments. Addressing these clinical challenges is of significant practical relevance. A study by Wenjiao Jin et al. demonstrated that inhibiting PCSK9 markedly enhances the efficacy of lenvatinib in hepatocellular carcinoma, suggesting synergistic potential [15]. Lenvatinib, a multi-target tyrosine kinase inhibitor, plays a critical role in treating advanced TCs, including ATC [20, 21]. Therefore, investigating PCSK9’s role and mechanism in ATC could have meaningful clinical implications, especially in enhancing lenvatinib’s efficacy.

Additionally, PCSK9 inhibitors are already well-developed and approved for clinical use, offering a practical therapeutic option. For locally advanced TC with strong PD-L1 expression, particularly in ATC cases, appropriate inhibitors remain essential. Liu et al. showed that PCSK9 inhibition enhances the efficacy of immune checkpoint blockade therapy, further motivating investigations into its therapeutic potential in ATC. Given the common resistance to monotherapy in ATC, exploring combination therapies could provide broader clinical benefits.

In this study, we focused on PCSK9’s role in ATC development. We found that PCSK9 overexpression correlates with poor prognosis in patients with ATC. Both genetic and pharmacological inhibition of PCSK9 considerably reverse its oncogenic effects in vitro and in vivo. Proteomic analysis revealed that PCSK9 drives ATC cell proliferation and invasion by inhibiting E-cadherin expression. Mechanistic studies indicated that PCSK9 promotes E-cadherin degradation through the lysosomal pathway. Furthermore, the loss of p53 function—a hallmark of ATC—de-repressed *PCSK9* transcriptionally.

Our findings underscored the critical role of PCSK9 in regulating E-cadherin degradation and suggest that PCSK9 could serve as a novel therapeutic target for ATC, with implications for expanding the therapeutic landscape for this aggressive cancer.

## Methods

### Tissue samples and cell lines

Tissue samples from patients diagnosed with thyroid cancer were collected at the Fudan University Shanghai Cancer Center (FUSCC, Shanghai, China) between January 2010 and December 2019. The Ethics Committee of the Fudan University Shanghai Cancer Center approved this study, and all participating patients provided informed consent and authorization (approval number: 050432-4-2307E). Human anaplastic thyroid cancer (ATC) cell lines (KHM-5M, CAL62, SW1736, and C643), papillary thyroid cancer (PTC) cell lines (TPC1, BCPAP, and K1), and follicular thyroid cancer (FTC) cell lines (FTC133) were acquired from the Cell Bank of the Committee for Conservation of Typical Cultures of the Chinese Academy of Sciences. KHM-5M, CAL62, and C643 cells were maintained in Dulbecco’s Modified Eagle Medium (HyClone, Logan, UT, USA); TPC1, BCPAP, FTC133, and SW1736 cells were maintained in Roswell Park Memorial Institute (RPMI) 1640 medium (HyClone, Logan, UT, USA); while K1 cells were maintained in F12K medium (Gibco, New York, USA), supplemented with 10% fetal bovine serum (FBS) and 100 IU/mL of penicillin and streptomycin (Gibco, New York, USA).

### External RNA-seq data acquisition and processing

The RNA-seq data of thyroid cancer were obtained from the GEO databases, including GSE76039 (20 ATC, 17 PDTC), GSE65144 (12 ATC, 13 Normal), GSE53157 (4 FTC, 8 FVPTC, 5 PDTC, 7 PTC, 3 Normal), GSE33630 (11 ATC, 49 PTC, 45 Normal) and GSE29265 (9 ATC, 20 PTC, 20 Normal) with consistent chip platform (Affymetrix Human Genome U133 Plus 2.0 Array). For data preprocessing, the R package affy was used to apply the rma method for background correction and normalization, using default parameters. Principal component analysis (PCA) was performed with the prcomp function to assess the integration of the datasets. Both primary and recurrent samples were included in the comparison of mRNA expression among different pathological types.

### Gene set enrichment analysis (GSEA)

A total of 52 ATC samples were divided into high and low PCSK9 subgroups based on median expression levels. Differentially expressed genes (DEGs) were identified using the limma method with the diff_analysis function from the easy TCGA package. DEGs with a *p* value < 0.05 were selected for GSEA, utilizing the clusterProfiler package and based on the HALLMARK gene set.

### Reagents and antibodies

The following antibodies were used for the western blot analysis: PCSK9 (A25215 ABclonal, Wuhan, China), GAPDH (60004-1-Ig Proteintech, Wuhan, China), Actin (66009-1-Ig Proteintech, Wuhan, China), E-cadherin (610404 BD, New York, USA), ATP1A1 (14418-1-AP Proteintech, Wuhan, China), LAMP2 (66301-1-Ig Proteintech, Wuhan, China), N-cadherin (22018-1-AP Proteintech, Wuhan, China), Vimentin (10366-1-AP Proteintech, Wuhan, China), NIS (24324-1-AP Proteintech, Wuhan, China), TG (ab156008 Abcam, Boston, USA), TSHR (14450-1-AP Proteintech, Wuhan, China), MYC (16286-1-AP Proteintech, Wuhan, China), FLAG (20543-1-AP Proteintech, Wuhan, China), and p53 (9282 Cell Signaling Technology, Danvers, USA).

### Knockout and overexpression of PCSK9 in ATC cell lines

To knock out the PCSK9 gene, we cloned the following sgRNA sequences: sg1:5ʹ- CTCCTCGATGTAGTCGACAT-3ʹ and sg2:5ʹ- CTCGGGCACATTCTCGAAGT-3ʹ, and inserted them into the LentiCRISRPv2 vector (Addgene ID: #52961). 293 T cells were used to package the lentivirus-sgRNA (PCSK9) along with the packaging vector. The PCSK9/E-cadherin overexpression lentiviral construction systems were obtained from GeneChem (Shanghai, China). Following the manufacturer’s guidelines, stable transfections were performed using puromycin (2 μg/mL) for 7 days.

### Cell membrane and lysosome isolation and extraction

Cell membrane isolation and extraction were conducted using the MinuteTM Plasma Membrane Protein Isolation and Cell Fractionation Kit (SM-005 Invent Biotechnologies, Plymouth, MN, USA). Cells were washed with pre-cooled PBS, incubated with 500 μl Buffer A on ice for 5–10 min, and then centrifuged at 16,000 × *g* for 30 s. The pellet was resuspended, followed by centrifugation at 700 × *g* for 1 min to obtain a mixture of plasma membrane and cell organelles. After resuspending the pellet in 200 μl Buffer B and centrifugation at 700 × *g* for 5 min, the supernatant was collected and transferred to a 2 ml centrifuge tube. Addition of 1.6 ml PBS, gentle mixing, and centrifugation at 16,000 × *g* for at least 30 min yielded the plasma membrane pellet after removal of the supernatant.

As for lysosome, the isolation and extraction were conducted with MinuteTM Lysosome Isolation Kit (LY-034 Invent Biotechnologies, Plymouth, MN, USA). After washing cells with pre-chilled PBS, 500 μl of Buffer A was added and incubated on ice for 5–10 min. The mixture was centrifuged at 16,000 × *g* for 30 s, and the pellet was resuspended in the receiving tube. Centrifugation at 2000 × *g* for 3 min yielded a pellet containing cell nuclei, large debris, and intact cells. The supernatant was transferred to a new tube and centrifuged at 11,000 × *g* for 15 min. After resuspending the pellet in 200 μl Buffer A and vortexing, centrifugation at 2000 × *g* for 4 min was performed. The supernatant was transferred to a new tube, 100 μl Buffer B was added, and the mixture was briefly vortexed. After incubating on ice for 30 min and centrifuging at 11,000 × *g* for 10 min, the supernatant was completely removed. Centrifugation for a few seconds at 11,000 × *g* removed residual liquid, yielding a pellet highly enriched in lysosomal components.

### Western blotting

Western blotting was conducted as follows: cultured cells were rinsed with ice-cold phosphate-buffered saline (PBS), and total cell protein lysates were extracted at 4 °C with radioimmunoprecipitation assay (RIPA) lysis buffer (Beyotime, Shanghai, China) supplemented with a 1% protease inhibitor cocktail (MedChemExpress, New Jersey, USA). Following centrifugation at 12,000 × *g* for 20 min at 4 °C, the supernatant was collected and mixed with loading buffer. The samples were separated by 10% sodium dodecyl sulfate-polyacrylamide gel electrophoresis (SDS-PAGE) and transferred onto polyvinylidene fluoride (PVDF) membranes. After blocking with 5% skimmed milk for 2 h at room temperature, the PVDF membrane was incubated with primary antibodies overnight at 4 °C. Subsequently, the PVDF membrane was washed with Tris-buffered saline and incubated with secondary antibodies. Finally, the membranes were visualized using an enhanced chemiluminescence reagent (Beyotime, Shanghai, China). The dilution ratios of antibodies are as follows: Actin (1:5,000), GAPDH (1:5,000), PCSK9 (1:1,000), E-cadherin (1:1,000), LAMP2 (1:1,000), ATP1A1 (1:2,000), N-cadherin (1:1,000), Vimentin (1:1,000), NIS (1:1,000), TG (1:2,000), TSHR (1:1,000), MYC (1:2,000), FLAG (1:20,000), and p53 (1:1,000). Secondary antibodies included anti-rabbit (1:3,000) and anti-mouse (1:3,000).

### Total RNA extraction and qRT-PCR

Total RNA was extracted using the TRIzol reagent (Invitrogen, Waltham, Massachusetts, USA) following the manufacturer’s protocol. RNA concentration was detected using a NanoDrop2000 spectrophotometer. Subsequently, 1 μg of total RNA was reverse-transcribed using a PrimeScript RT reagent kit (Takara, Dalian, China). cDNA was amplified using SYBR Green Premix Ex Taq (Takara, Dalian, China), following the manufacturer’s protocol. Gene expression levels were standardized against β-actin (mRNA) expression and determined using the comparative Ct (2-ΔΔCt) method. The primer sequences are listed in Table S1.

### Immunohistochemistry (IHC) assays

Following deparaffinization, the paraffin sections were treated with 3% hydrogen peroxide at 26 °C for 10 min to inhibit endogenous peroxidase activity. After blocking with 10% goat serum, the sections were incubated overnight at 4 °C with primary antibodies. Sections were then treated with rabbit secondary antibodies and stained with 3,3’-diaminobenzidine (DAB). An H-score was determined utilizing the subsequent formula: H-score = ∑(PI×I) = (percentage of cells of weak intensity ×1) + (percentage of cells of moderate intensity ×2) + (percentage of cells of strong intensity ×3). The H-score was documented as a continuous variable. The median expression level of PCSK9 was chosen as the cutoff for classification.

### Animal studies

To establish a nude mouse lung metastasis tumor model, CAL62-luc cells (10^6^ cells) were injected into the tail vein of female BABL/c nude mice (3–4 weeks old) obtained from GemPharmatech (Nanjing, China). To evaluate the antitumor effects of PF846 in metastasis assays, all mice were assigned randomly 1 week after the CAL62 injection to receive PF846 (50 mg/kg every 2 days) in a vehicle containing 200 mM citrate buffer in 0.5% methylcellulose (w/v) by oral gavage. As the control group, six additional mice received 200 mM citrate buffer in 0.5% methylcellulose (w/v) by oral gavage. The IVIS Spectrum animal imaging system (PerkinElmer) was used to monitor the metastatic loci formed by CAL62 cells over 6 weeks. Each mouse was administered 100 μL of Xeno Light D-luciferin Potassium Salt (15 mg/mL; Perkin Elmer). The survival times of the mice were recorded. Approximately 6 weeks after treatment, all mice were euthanized, and lung metastases were analyzed.

For the subcutaneous tumor model, CAL62 cells (5 × 10^6^ cells) were subcutaneously injected into the right dorsal flank of six-week-old female BALB/c nude mice. After injection of CAL62 cells for 2 weeks, the mice were randomly assigned to two subgroups (*n* = 6 per subgroup) that were treated with PBS control or PF846 (50 mg/kg, per 2 days, p.o.). The long (L) and short (S) diameters of the tumors were measured every 4 days. The tumor volume was calculated using the formula: volume = (L * W^2) / 2. All mice were euthanized 3–4 weeks after the injection of CAL62 cells. Mice were euthanized if any tumor dimension reached 20 mm, tumor volume exceeded 2000 mm^3^, or if a mouse experienced a 20% reduction in body weight. All experiments were conducted under the guidance of the FUSCC Animal Experimentation Ethics Committee.

### Assessment of cell proliferation, colony formation, and invasion abilities

To assess cell proliferation, 2 × 10^3^ cells were plated in each well of a 96-well plate and cultured for the indicated time. Following this, 10 μL of Cell Counting Kit-8 (CCK-8) reagent (Dojindo Molecular Technologies, Kumamoto, Japan) was added into each well and incubated for an additional hour. The absorbance per well at a 450 nm wavelength (OD450) was assessed and analyzed.

To assess the clone formation capability, 500 cells were plated into each well of a 6-well plate and allowed to grow for approximately 1 week. Upon reaching a cell count of over 50 per clone, the cells were stained with 0.2% crystal violet for 30 min. After washing three times with PBS, the clones were photographed and quantified.

To evaluate cell invasion capacity, 4 × 10^4^ cells were suspended in a 200 μL culture medium. Subsequently, the cells were seeded into the upper chamber of Transwell plates (BD Biosciences, Bedford, MA, USA) pre-coated with 50 μL of Matrigel (BD Biosciences). At the same time, 600 μL of culture medium containing 10% FBS was added into the bottom chamber. After a 24-h incubation at 37 °C, the Transwell plates were treated with 4% paraformaldehyde for 30 min to fix the cells, followed by staining with 0.25% crystal violet for another 30 min. After removing the cells from the upper chamber, invasive cells were photographed and quantified.

### Proteomic analysis of thyroid cancer cells upon PCSK9 overexpression

PCSK9-overexpressing and control cells were washed three times with PBS buffer to remove any residual medium and serum. Cells were lysed in lysis buffer containing protease and phosphatase inhibitors for 30 min, followed by centrifugation at 14,000 × *g* for 15 min. The supernatant was collected, and protein concentration was measured. Extracts (1 mg of protein) were reduced with 10 mM dithiothreitol at 56 °C for 30 min and alkylated with 10 mM iodoacetamide in the dark at room temperature for 30 min. Samples were then digested with trypsin using a filter-aided sample preparation method, vacuum-dried, and prepared for mass spectrometry analysis in Orbitrap Fusion Lumos Hybrid Quadrupole-Orbitrap mass spectrometer (Thermo Fisher Scientific). Dried peptide samples were re-dissolved in Solvent A (0.1% formic acid in water) and loaded onto a 2 cm self-packed trap column. Peptides were then separated on an analytical column using a 150-min gradient elution (Solvent A: 0.1% formic acid in water; Solvent B: 0.1% formic acid in 80% acetonitrile). Eluates were ionized at a voltage of 2 kV and introduced into the mass spectrometer, with data acquired in a data-dependent acquisition mode. Mass spectrometry data files were searched against a reference database to identify proteins, followed by both qualitative and quantitative analyses. This process included a comprehensive assessment of global protein expression differences and the identification of significantly differentially expressed proteins.

### Immunofluorescence

Briefly, the cells seeded on confocal dishes were fixed with 4% paraformaldehyde for 10 min, followed by permeabilization with 0.1% Triton X-100 for 5 min at room temperature. Then, the cells were incubated overnight at 4 °C with primary antibodies, followed by incubation with fluorescent secondary antibodies for 1 h at room temperature. After washing with PBS, cells were stained with 4′,6-diamidino-2-phenylindole (DAPI) for 10 min. Finally, observations were made and photographed using a confocal microscope.

### Chromatin immunoprecipitation assays

Chromatin immunoprecipitation (ChIP) assays were performed using a Simple ChIP Enzymatic Chromatin IP Kit (Magnetic Beads, Cell Signaling Technology, #9003). Briefly, KHM-5M and CAL62 cells were cross-linked with 1% formaldehyde at room temperature for 10 min. Following sonication, the chromatin DNA in the cell lysates was sheared into fragments ranging from 200 to 500 bp. Subsequently, the chromatin supernatant was incubated overnight at 4 °C with rotation, concomitant with an equivalent volume of either anti-p53 or anti-immunoglobulin (Ig) G antibody. Following de-crosslinking of the protein/DNA complexes, qRT-PCR was conducted employing specific primers (forward primer: 5ʹ-GGTAGGTGATCCCTGCGGA-3ʹ, reverse primer: 5ʹ-GCAGAGGGGACCATCCTCTT-3ʹ) to discern the immunoprecipitated DNA.

### Luciferase reporter assays

Luciferase reporter gene analysis was performed using a Dual-Luciferase Reporter Assay System (Promega, Madison, WI, USA). The PCSK9 promoter sequence and its mutated variants were inserted separately into the pGL3 basic vector. The constructed pGL3-PCSK9-promoter plasmids and the Renilla luciferase control plasmid were co-transfected into the cells. Luciferase activity was assessed 72 h post-transfection. The reporter gene activity was normalized to Renilla luciferase activity.

### Coimmunoprecipitation (Co-IP) assay

To investigate the interaction between endogenous PCSK9 and E-cadherin, CAL62 cell lysates were extracted using IP lysis buffer (Beyotime, Shanghai, China). These lysates were then incubated overnight at 4 °C with either 2 µg of anti-PCSK9 antibody or anti-E-cadherin antibody, with anti-rabbit IgG (Beyotime, Shanghai, China) as a control. Protein A/G magnetic beads (Beyotime, Shanghai, China) were added to the cell mixture and incubated for 4 h at 4 °C.

To study the interaction between exogenous PCSK9 and E-cadherin, CAL62 cells were transfected with the pCDNA3.1-PCSK9-Flag or pCDNA3.1-E-cadherin-Myc plasmid. After 48 h of culture, the cells were homogenized in a lysis buffer for immunoprecipitation (Beyotime, Shanghai, China). Subsequently, the cell lysates were incubated with 20 µL of anti-IgG magnetic beads (Beyotime, Shanghai, China) overnight at 4 °C. The following day, the mixtures were incubated with either anti-Flag or anti-Myc magnetic beads (Beyotime, Shanghai, China) for 4 h at 4 °C. Subsequently, the beads were washed four times with IP lysis buffer, and the precipitates were collected and eluted with 1× SDS sample buffer. Western blotting was performed to analyze the samples.

### Statistical analysis

All experimental data were analyzed using SPSS software (version 22.0; IBM Corp., Armonk, NY, USA) and visualized using GraphPad Prism software (version 8; GraphPad Software, La Jolla, CA, USA). The results are presented as mean ± standard deviation (mean ± SD). Statistical analysis between two groups was conducted using a *t* test, whereas Fisher’s least significant difference (LSD) test was employed following a one-way analysis of variance (ANOVA) for comparisons across multiple groups. Statistical significance was defined as *p* < 0.05. Each experiment was conducted independently at least three times.

## Results

### PCSK9 expression in ATC is associated with advanced tumor stage and metastasis

**Fig. 1:**
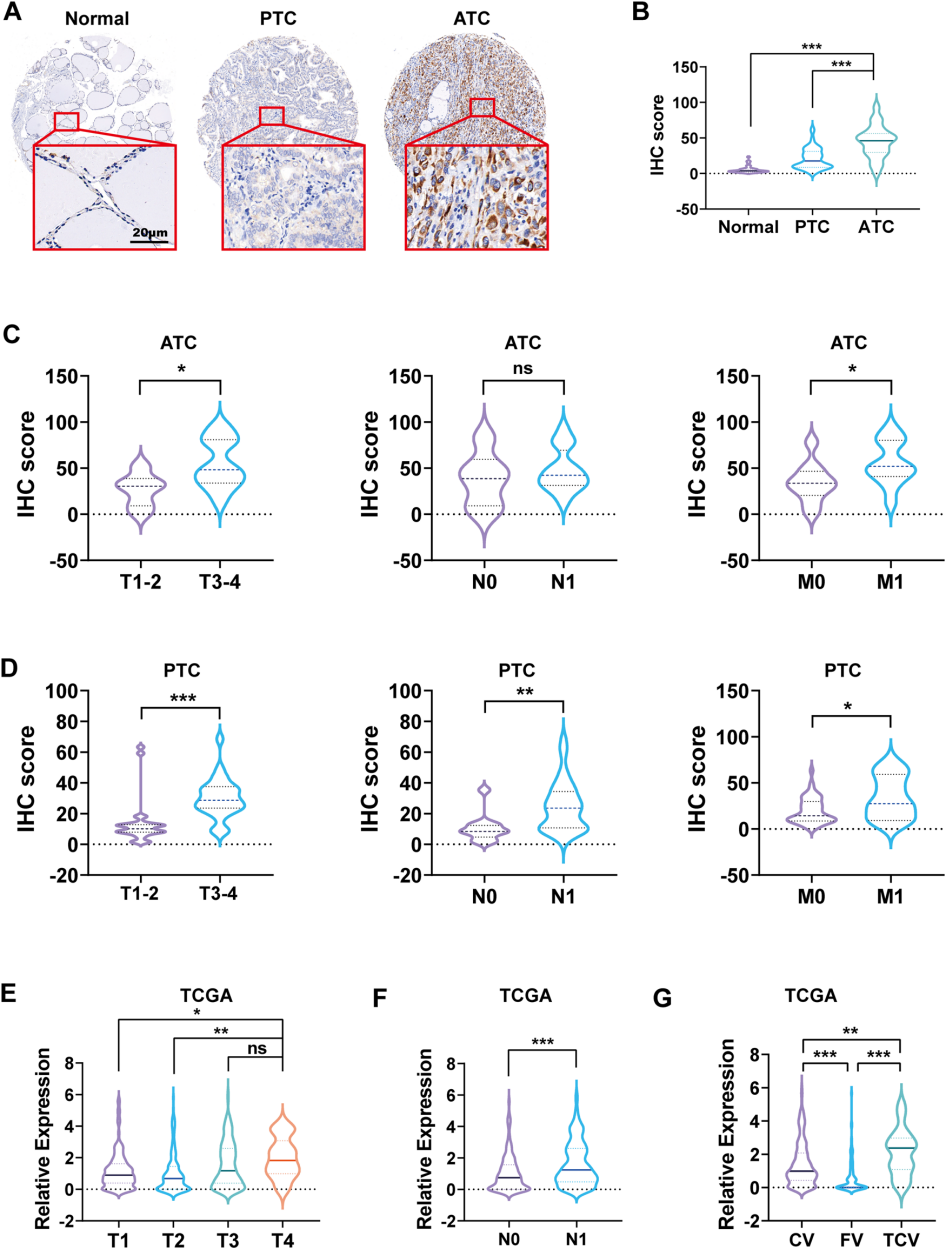
PCSK9 expression in ATC is associated with advanced tumor stage and metastasis. **A** Representative IHC images of PCSK9 protein expression in normal, PTC, and ATC tissues (normal: *n* = 44, PTC: *n* = 54, ATC: *n* = 22). Scale bar, 20 μm. **B** The expression of PCSK9 protein in normal, PTC and ATC tissues (normal: *n* = 44, PTC: *n* = 54, ATC: *n* = 22). **C** The expression of PCSK9 protein in different TNM stages of ATC was detected by IHC staining. (T1-2: *n* = 6, T3-4: *n* = 16, N0: *n* = 6, N1: *n* = 16, M0: *n* = 10, M1: *n* = 12). **D** The expression of PCSK9 protein in different TNM stages of PTC was detected by IHC staining. (T1-2: *n* = 27, T3-4: *n* = 27, N0: *n* = 11, N1: *n* = 43, M0: *n* = 47, M1: *n* = 7). **E** The expression of PCSK9 (mRNA) in different grades of thyroid cancer in TCGA. **F** The expression of PCSK9 (mRNA) in N0 or N1 of thyroid cancer in TCGA. **G** The expression of PCSK9 (mRNA) in classical, follicular, and tall cell variants thyroid cancer in TCGA. Comparisons among multiple groups were performed by one-way ANOVA followed by Fisher’s LSD test. (*p < 0.05, **p < 0.01, ***p < 0.001). PTC papillary thyroid cancer, ATC anaplastic thyroid cancer, IHC Immunohistochemistry, CV classical variant, FV follicular variant, TCV tall cell variant.

To elucidate the role of PCSK9 in TC, immunohistochemistry (IHC) was performed to evaluate its expression in PTC, ATC, and normal thyroid tissues from FUSCC. The correlation between PCSK9 expression levels and various clinicopathological features was analyzed (Table S2). PCSK9 expression was significantly elevated in PTC and ATC tissues compared to normal tissues. Notably, PCSK9 levels were markedly higher in ATC tissues than in PTC tissues (Fig. 1A, B).

Further analysis revealed that in patients with ATC, PCSK9 expression was significantly associated with higher pathological tumor stage (pT) and distant metastasis; however, no significant correlation was observed with higher lymph node metastasis grades (pN) (Fig. 1C). These findings align with the clinical characteristics of ATC, where patients often present with locally invasive tumors and early distant metastases at diagnosis.

In contrast, among patients with PTC, increased PCSK9 expression was closely linked to higher pT stage, pN grade, and distant metastasis, underscoring PCSK9’s role in driving tumor progression in TC (Fig. 1D). Owing to the extremely poor prognosis and short survival time associated with ATC, along with the relatively small sample size, the prognosis was analyzed separately for patients with PTC. Although no statistically significant difference was observed, patients with PTC with high PCSK9 expression tended to have worse overall outcomes (Fig. S1B).

Consistent findings were observed in TCGA data, which primarily included PTC and other differentiated TCs. Higher PCSK9 expression was strongly correlated with pT or pN stage (Fig. 1E, F). Additionally, PCSK9 levels were significantly elevated in the tall cell variant (TCV) of TC compared to the classical (CV) and follicular variants (FV). This aligns with the typically poor prognosis of patients with the TCV (Fig. 1G). To further validate these findings, RNA-seq data from the GEO database were analyzed, encompassing 227 cases of TC across different pathological types. PCSK9 expression was significantly upregulated in ATC tissues compared to PTC, poorly differentiated thyroid cancer (PDTC), and normal thyroid tissues (Fig. S1A).

### PCSK9 drives the malignant phenomenon of ATC

Next, we examined the expression of PCSK9 in various TC cell lines. Notably, PCSK9 was significantly overexpressed in ATC cell lines, particularly KHM-5M and CAL62, compared to PTC cell lines (TPC1, K1, and BCPAP) and the FTC cell line (FTC133) (Fig. S1C). Furthermore, Gene Set Enrichment Analysis revealed that elevated PCSK9 expression is linked to several tumor proliferation and development pathways, including Wnt/β-catenin, hypoxia, p53, and epithelial-mesenchymal transition (EMT) pathways (Fig. S1D).

**Fig. 2:**
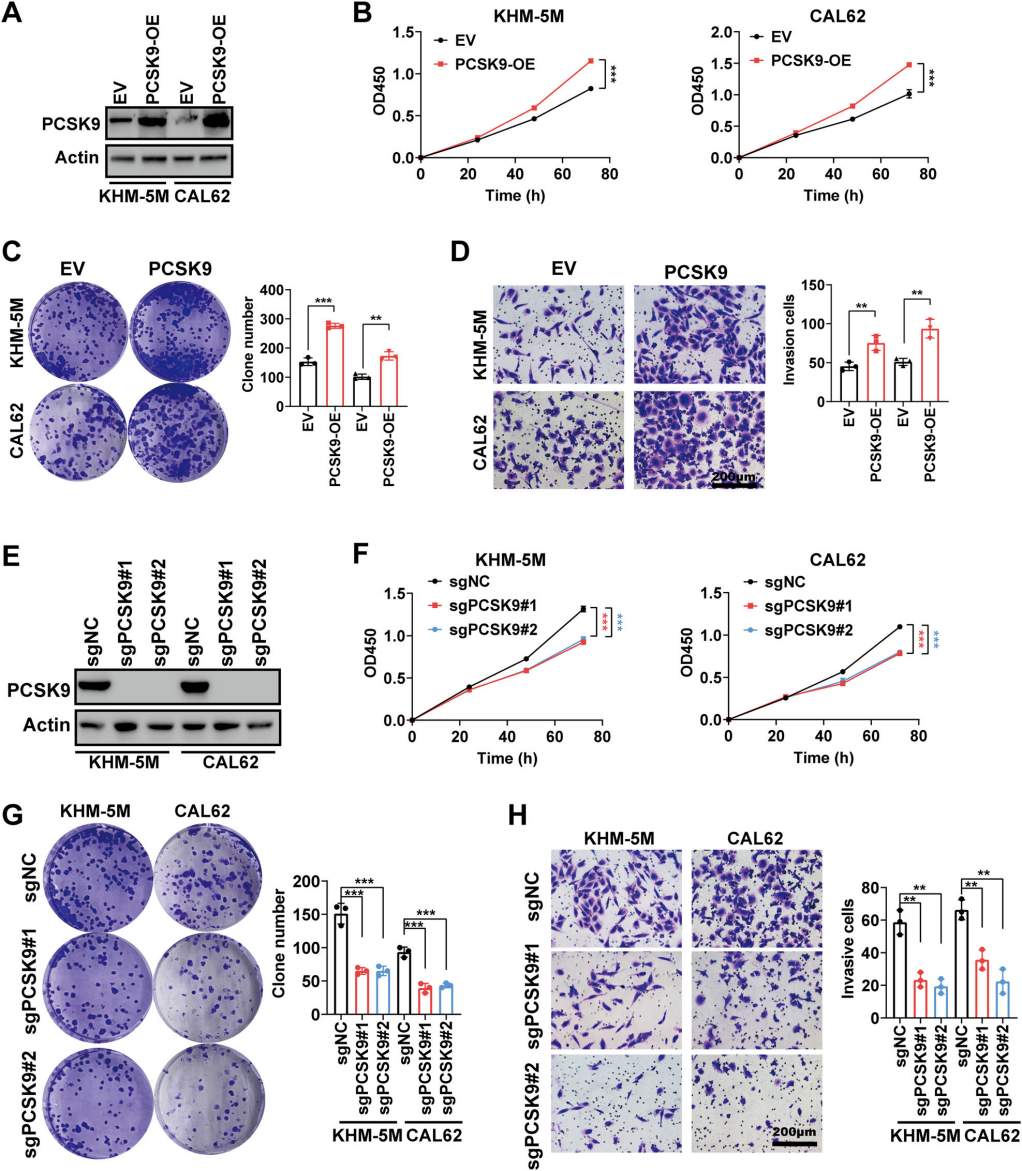
PCSK9 drives malignant phenomenon of ATC. **A** Overexpression of PCSK9 validated by Western Blotting analysis in KHM-5M and CAL62 cells (EV: Empty vector; OE: Overexpression). **B** The proliferative abilities of EV and PCSK9-OE detected by CCK8 assay in KHM-5M and CAL62 cells. **C** The clone formation abilities assessed in KHM-5M and CAL62 cells upon PCSK9 overexpression. **D** The invasive abilities of KHM-5M and CAL62 cells detected by Transwell assay upon PCSK9 overexpression. Scale bar, 200 μm. **E** Knockout of PCSK9 in KHM-5M and CAL62 cells validated by Western Blotting analysis. **F** The proliferative abilities of PCSK9 knockout ATC cells detected with CCK8 assay. **G** The clone formation abilities of PCSK9 knockout ATC cells. **H** The invasive abilities of PCSK9 knockout ATC cells evaluated with Transwell assay. Scale bar, 200 mm. The data are presented as mean ± SD. Statistical analysis of the data from 2 groups was conducted with Student’s *t* test. Comparisons among multiple groups were conducted with one-way ANOVA followed by Fisher’s LSD test. (**p < 0.01, ***p < 0.001). PCSK Proprotein Convertase Subtilisin/Kexin Type 9, EV empty vector, OE overexpression, sgPCSK9 single guide PCSK9.

To explore the role of PCSK9 in ATC progression, we overexpressed or knocked out PCSK9 in KHM-5M and CAL62 cells (Fig. 2A, E). PCSK9 overexpression significantly promoted the proliferation and colony formation of KHM-5M and CAL62 cells (Fig. 2B, C). Additionally, overexpression of PCSK9 enhanced the invasive ability of these cells (Fig. 2D). In contrast, PCSK9 knockout significantly inhibited both the proliferation and colony formation abilities of KHM-5M and CAL62 cells (Fig. 2F, G). Moreover, PCSK9 knockout suppressed the invasiveness of these cells (Fig. 2H).

Based on public databases and IHC experiments showing that PCSK9 expression levels are significantly higher in ATC than in PTC, we hypothesized that PCSK9 may play a role in the dedifferentiation process of TC. Preliminary experiments supported our hypothesis that overexpression of PCSK9 in PTC cell lines (TPC1 and BCPAP) significantly suppressed the expression of thyroid differentiation markers (NIS, TG, and TSHR) (Fig. S2A). Furthermore, overexpression of PCSK9 markedly promoted the proliferation and invasion of TPC1 and BCPAP cells (Fig. S2B, C).

### E-cadherin is essential for the PCSK9-driven malignant phenomenon of ATC

**Fig. 3:**
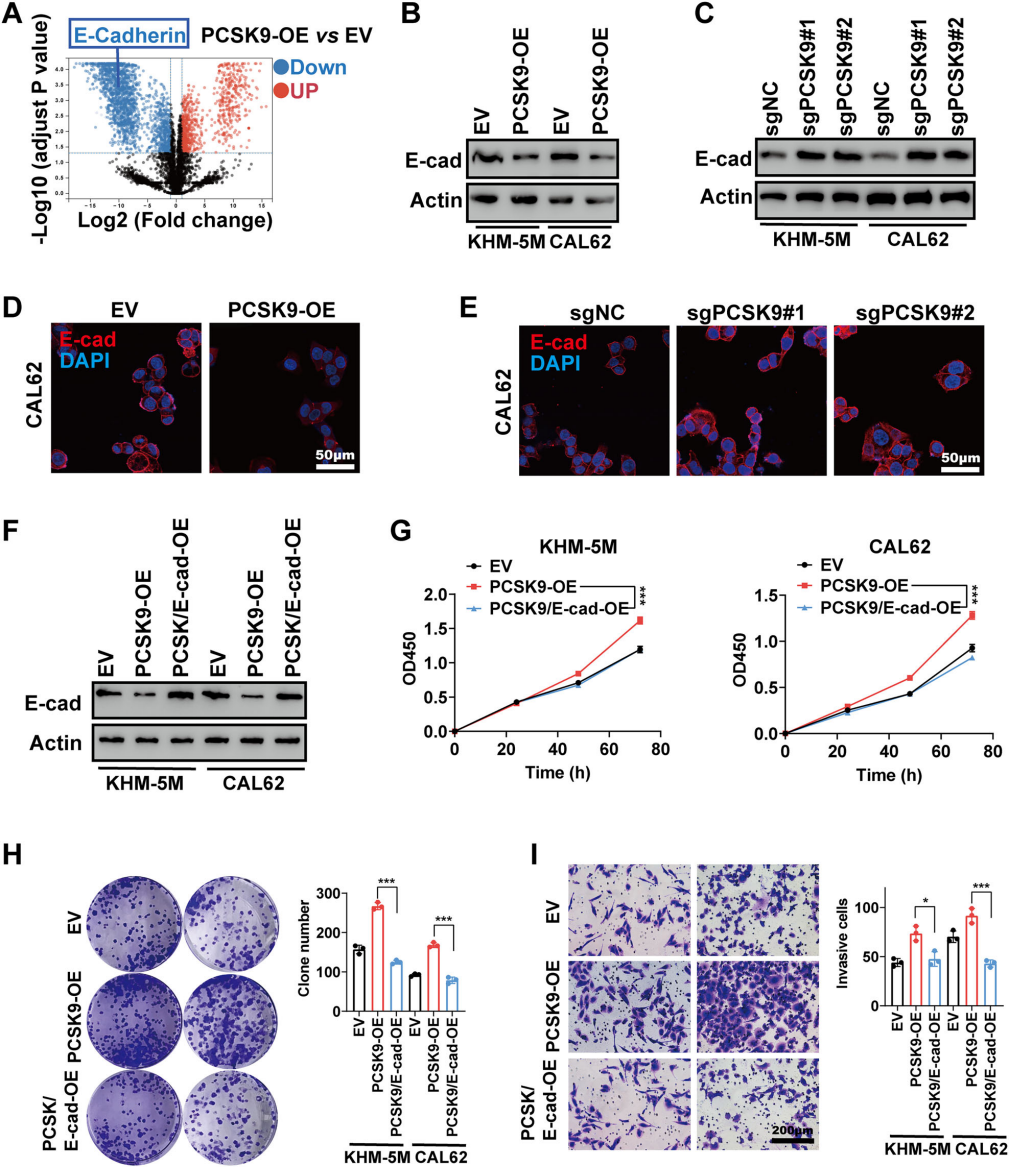
E-cadherin is essential for PCSK9 driven malignant phenomenon of ATC. **A** The proteomic analysis results of EV and PCSK9 overexpressing CAL62 cells. **B** Overexpression of PCSK9 repressed the protein level of E-cadherin in KHM-5M and CAL62 cells. **C** Knockout of PCSK9 increased the protein level of E-cadherin in KHM-5M and CAL62 cells. **D** Immunofluorescence staining revealed that PCSK9 overexpression inhibited the expression of E-cadherin on the cell membrane. **E** PCSK9 Knockout increased the expression of E-cadherin on the cell membrane. **F** Simultaneously overexpression of PCSK9 and E-cadherin in KHM-5M and CAL62 validated by Western Blotting analysis. **G** Overexpression of E-cadherin counteracted the promotion of PCSK9 overexpression on the proliferation of KHM-5M and CAL62 cells detected with CCK8 assay. **H** Overexpression of E-cadherin counteracted the promotion of clone formation upon PCSK9 overexpression of KHM-5M and CAL62 cells. **I** The promotion of PCSK9 overexpression on invasion in ATC was counteracted by E-cadherin overexpression detected with Transwell assay. Statistical analysis of the data from 2 groups was conducted with Student’s t test. Comparisons among multiple groups were conducted with one-way ANOVA followed by Fisher’s LSD test. (*p < 0.05, ***p < 0.001). E-cad E-cadherin.

We further investigated the mechanism by which PCSK9 promotes ATC progression. A proteomic analysis of EV and PCSK9-overexpressing (PCSK9-OE) CAL62 cells revealed a significant downregulation of E-cadherin at the protein level upon PCSK9 overexpression (Fig. 3A). E-cadherin is a critical protein involved in maintaining cell-cell adhesion and tissue structure, and it plays a key role in tumorigenesis and metastasis [22]. Therefore, we examined the expression of E-cadherin in ATC cells and found that PCSK9 overexpression significantly inhibited E-cadherin protein levels; in contrast, PCSK9 knockout significantly upregulated E-cadherin protein levels in KHM-5M and CAL62 cells (Fig. 3B, C).

Additionally, as an essential protein for cell membrane adhesion, changes in E-cadherin expression were clearly observed in immunofluorescence experiments. We further confirmed the inhibitory effect of PCSK9 on E-cadherin protein levels by immunofluorescence staining of CAL62 cells (Fig. 3D, E). To determine if E-cadherin is a downstream target in the PCSK9-mediated promotion of ATC progression, we restored E-cadherin expression in PCSK9-OE KHM-5M and CAL62 cells (Fig. 3F). The results showed that restoring E-cadherin expression counteracted the effect of PCSK9 overexpression on the proliferation and colony formation of KHM-5M and CAL62 cells (Fig. 3G, H). Moreover, the promotion of invasion by PCSK9 overexpression was also counteracted by the restoration of E-cadherin expression (Fig. 3I).

Additionally, we assessed several critical EMT markers, as the loss of E-cadherin is a key driver of EMT. Consistent with our expectations, PCSK9 knockout inhibited the expression of N-cadherin and Vimentin, indicating that PCSK9 induces EMT (Fig. S1E).

### PCSK9 promotes endocytosis of E-cadherin through the lysosomal pathway

**Fig. 4:**
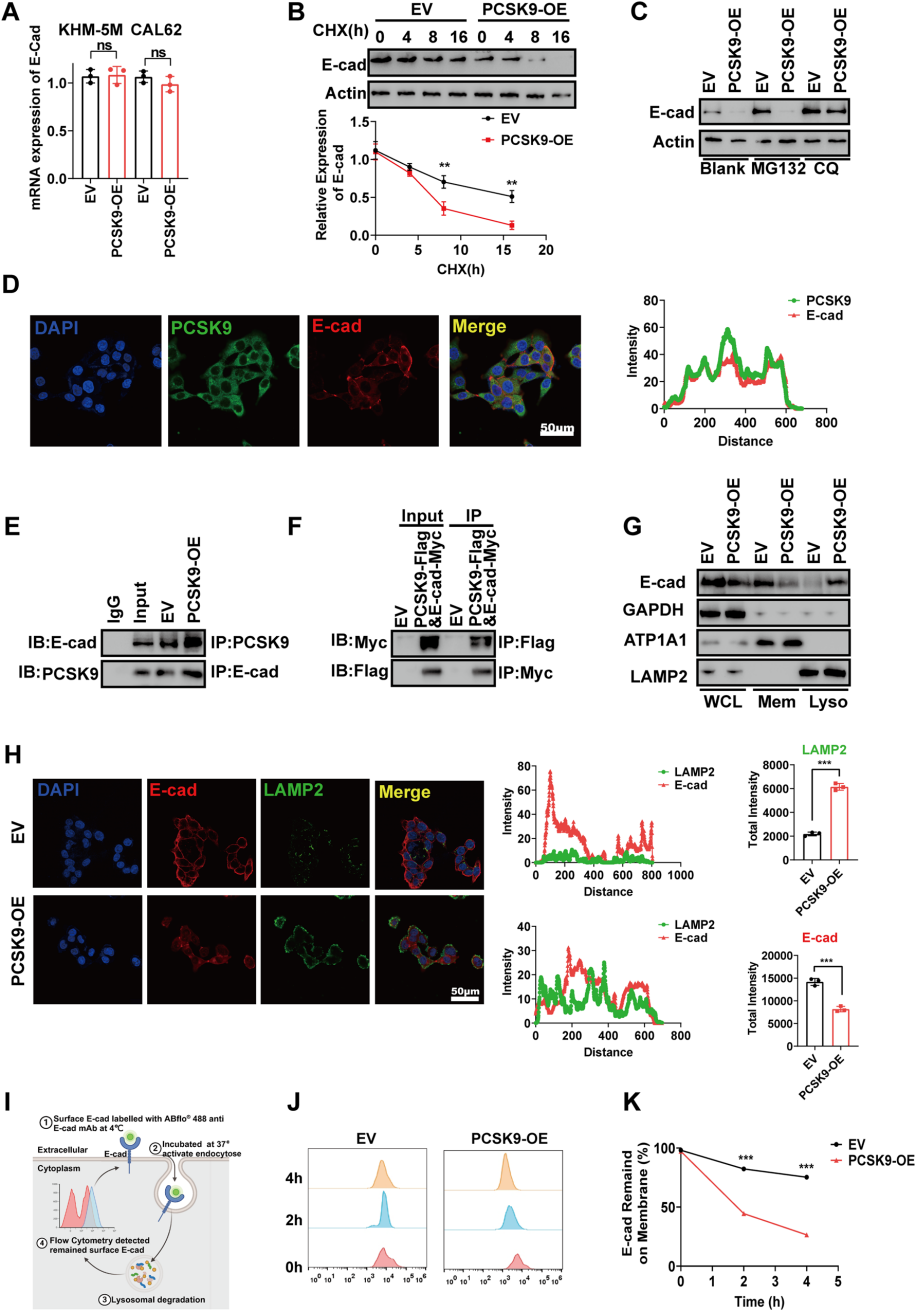
PCSK9 promotes endocytosis of E-cadherin through lysosomal pathway. **A** PCSK9 overexpression did not affect the mRNA expression of E-cadherin in KHM-5M and CAL62 detected in qPCR. **B** Overexpression of PCSK9 decreased the protein stability of E-cadherin. Protein synthesis inhibitor Cycloheximide (CHX, 50 μg/mL). **C** Overexpression of PCSK9 enhanced the lysosomal degradation of E-cadherin. Proteasome inhibitor MG132 (25 μM, 6 h), lysosomal inhibitor CQ (50 μM, 3 h). **D** Immunofluorescence staining of PCSK9 and E-cadherin in CAL62 cells. The colocalization analysis of PCSK9 and E-cadherin. Scale bar, 50 μm. **E**, **F** Interaction between PCSK9 and E-cadherin validated by Co-IP assays. **G** Protein levels of E-cadherin, ATP1A1 and LAMP2 in WCL, Mem and Lyso of EV and PCSK9-OE CAL62 cells. WCL: whole cell; Mem: membrane; Lyso: lysosome. **H** Immunofluorescence revealed the decreased protein levels of E-cadherin and LAMP2 in CAL62 cells upon PCSK9 overexpression. The colocalization analysis of E-cadherin and LAMP2. Scale bar, 50 μm. **I** A schematic diagram illustrating the experimental procedure of surface E-cadherin fluorescently labeled and detecting the remained surface E-cadherin after endocytosis and lysosomal degradation upon PCSK9 overexpression by flow cytometry. **J**, **K** The fluorescence intensity was measured at 0, 2, and 4 h after labeling, and quantitative analysis was conducted between EV and PCSK9-OE cells. Statistical analysis of the data from 2 groups was conducted with Student’s *t* test. Comparisons among multiple groups were conducted with one-way ANOVA followed by Fisher’s LSD test. (**p < 0.01, ***p < 0.001). CHX cycloheximide, ATP1A1 ATPase Na + /K+ transporting subunit alpha 1, LAMP2 lysosome-associated membrane glycoprotein 2, WCL whole cell, Mem membrane, Lyso lysosome.

Given the significant inhibition of E-cadherin expression following PCSK9 overexpression, we further explored the regulatory mechanism. We observed that PCSK9 overexpression did not affect E-cadherin mRNA expression (Fig. 4A). Moreover, there was no significant correlation between PCSK9 and E-cadherin mRNA in TC samples from the TCGA database, suggesting that PCSK9 regulates E-cadherin at the protein level rather than at the transcriptional level (Fig. S1F).

To investigate the stability of E-cadherin, we treated CAL62 cells with the protein synthesis inhibitor cycloheximide for 0–16 h. PCSK9 overexpression decreased E-cadherin stability; in contrast, PCSK9 knockout enhanced it (Fig. 4B, S1G). Given the key role of the ubiquitin-proteasome system in protein stability and degradation, along with the previously reported role of PCSK9 in promoting LDLR degradation via the lysosomal pathway [23], we further investigated the ubiquitination and lysosomal degradation of E-cadherin in the presence or absence of the proteasome inhibitor MG132 and the lysosomal inhibitor CQ. The results showed that MG132 failed to block the inhibitory effect of PCSK9 overexpression on E-cadherin protein levels; in contrast, CQ significantly suppressed this effect, promoting E-cadherin protein stability (Fig. 4C). These findings suggest that PCSK9 inhibits E-cadherin protein levels by promoting degradation via the lysosomal pathway.

We also confirmed the colocalization of PCSK9 and E-cadherin in the membranes of KHM-5M cells (Fig. 4D). Co-immunoprecipitation experiments further validated the interaction between both endogenous and exogenous PCSK9 with E-Cadherin in CAL62 cells using anti-PCSK9/E-cadherin or anti-Flag/Myc antibodies (Fig. 4E, F). To clarify the mechanism by which PCSK9 mediates the degradation of E-cadherin through the lysosomal pathway, we extracted proteins from the whole cell lysates (WCL), membranes, and lysosomes of CAL62 cells. The results showed that PCSK9 overexpression led to a significant decrease in E-cadherin protein levels in both the WCL and membrane fractions; in contrast, PCSK9 knockout increased E-cadherin expression. Notably, the protein level of E-cadherin increased considerably in the lysosome fraction upon PCSK9 overexpression; in contrast, it decreased considerably following PCSK9 knockout. (Fig. 4G, S1H). These data suggest that PCSK9 overexpression promotes the entry of E-cadherin into lysosomes for degradation, resulting in reduced levels of E-cadherin on the cell membrane.

Additionally, immunoblotting and cell immunofluorescence experiments revealed that PCSK9 overexpression decreased E-cadherin protein levels but increased lysosome-associated membrane protein 2 (LAMP2), which is involved in the lysosomal degradation pathway (Fig. 4G, H). Moreover, E-cadherin and LAMP2 showed increased colocalization following PCSK9 overexpression (Fig. 4H). We also fluorescently labeled E-cadherin on the cell membranes to detect the remaining E-cadherin undergoing lysosomal degradation after endocytosis (Fig. 4I). The fluorescence intensity of E-cadherin was measured at 0, 2, and 4 h post-labeling, revealing a significant decrease in the amount of remaining E-cadherin on the cell membrane after PCSK9 overexpression (Fig. 4J, K).

### Loss of p53 function derepresses *PCSK9* at the transcriptional level

**Fig. 5:**
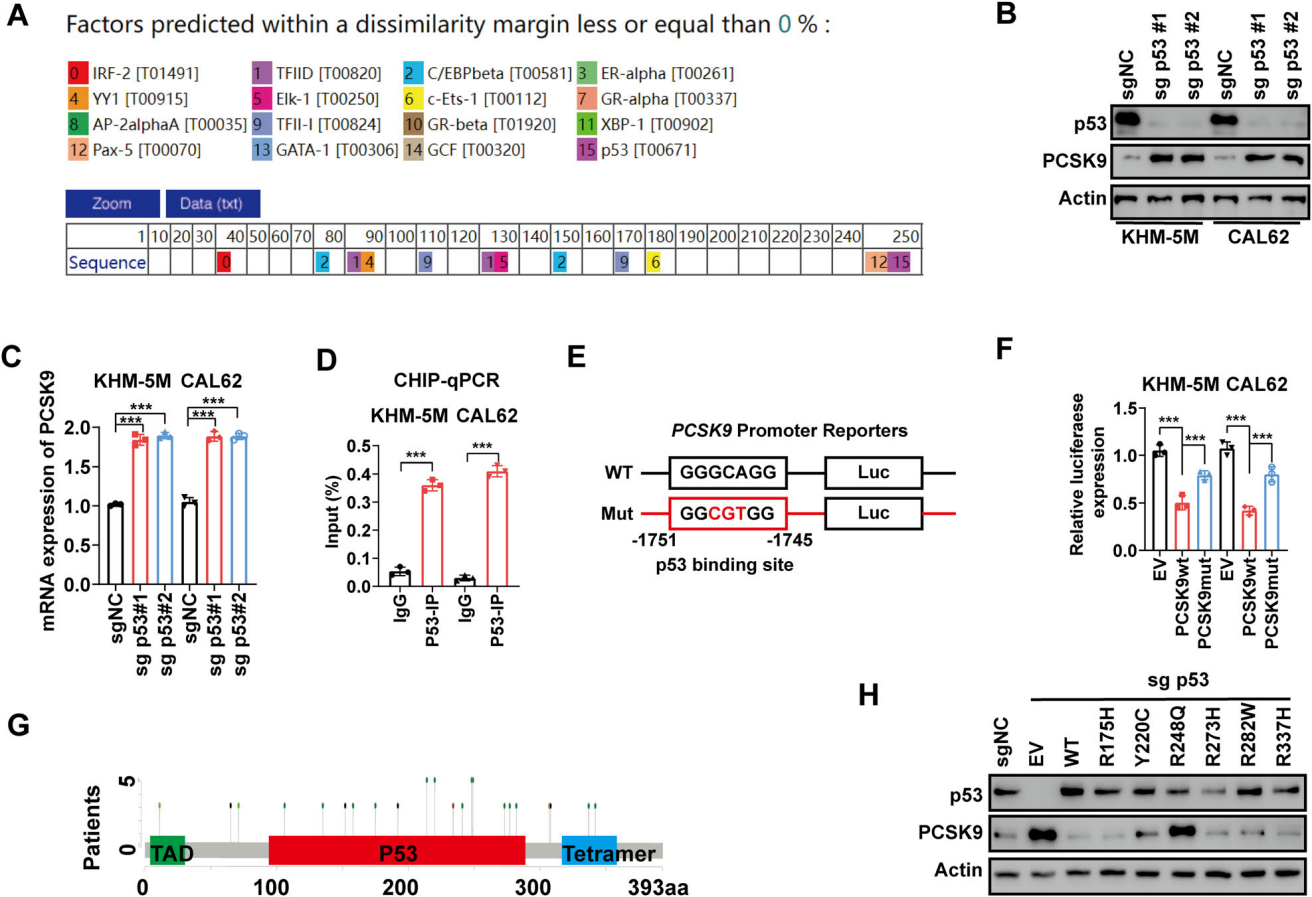
Loss of p53 function derepresses *PCSK9* at the transcriptional level. **A** Transcription regulatory elements prediction analysis of *PCSK9* promoter through PROMO database. **B** Knockout of p53 increased the protein level of PCSK9 in KHM-5M and CAL62 cells. **C** Upregulation of PCSK9 expression at the mRNA level following p53 knockout. **D** ChIP-qPCR assays confirmed the binding of p53 on *PCSK9* promoter. **E** Schematic of the predicted p53 binding site in the *PCSK9* promoter and the corresponding mutation sequences using JASPAR website (https://jaspar.elixir.no/). **F** Luciferase promoter activity analysis of *PCSK9* transcriptional activity between *PCSK9* wild type (PCSK9wt) and *PCSK9* p53 binding site mutated (*PCSK9*mut) vectors. **G** Diagrams showing common mutation locations of p53 in ATC patients. **H** Endogenous p53 was depleted. Empty vector (EV) and different p53 mutated vectors were transfected to p53 depleted cells. Statistical analysis of the data from 2 groups was conducted with Student’s t test. Comparisons among multiple groups were conducted with one-way ANOVA followed by Fisher’s LSD test. (**p < 0.01, ***p < 0.001).

PCSK9 upregulation has been observed in various solid tumors; however, the exact mechanisms remain incompletely understood. In ATC, p53 mutations are frequent and have garnered significant attention for their role in the dedifferentiation process. However, the specific mechanisms underlying these effects remain unclear. Through transcription regulatory element prediction using the PROMO database, we identified a critical p53 binding site in the promoter region of *PCSK9* (Fig. 5A).

To further investigate the role of p53 in regulating PCSK9, we examined its expression in ATC cells and found that p53 knockout significantly upregulated the protein levels of PCSK9 (Fig. 5B). At the mRNA level, we also observed increased *PCSK9* expression following p53 knockout (Fig. 5C). This prompted us to explore the role of p53 as a transcriptional repressor of *PCSK9*. ChIP-qPCR analysis confirmed that p53 was enriched in the *PCSK9* promoter region (Fig. 5D). Further investigation using the JASPAR database identified a p53 binding region (-1751 to -1745 bp from the transcription start site of *PCSK9*) (Fig. 5E).

To validate this interaction, we performed dual-luciferase reporter assays, which confirmed that mutation of the p53 binding sites impaired the transcriptional activation of *PCSK9* (Fig. 5F). Given that missense mutations are the most common form of p53 mutation in tumors, affecting its stability and transcriptional activity, we selected six of the most common mutation sites—R175H, Y220C, R248Q, R273H, R282W, and R337H—for further analysis [24] (Fig. 5G).

In p53-depleted cells, we restored p53 wild type and mutant p53 vectors. We found that plasmids with most mutation sites restored p53’s ability to suppress *PCSK9* expression; in contrast, the R248Q mutation prevented p53 from repressing *PCSK9* expression. This suggests that the R248Q mutation may play a critical role in disrupting p53’s regulation of *PCSK9* (Fig. 5H).

### PCSK9/E-cadherin axis regulates malignant phenomena of ATC through intracellular signaling

**Fig. 6:**
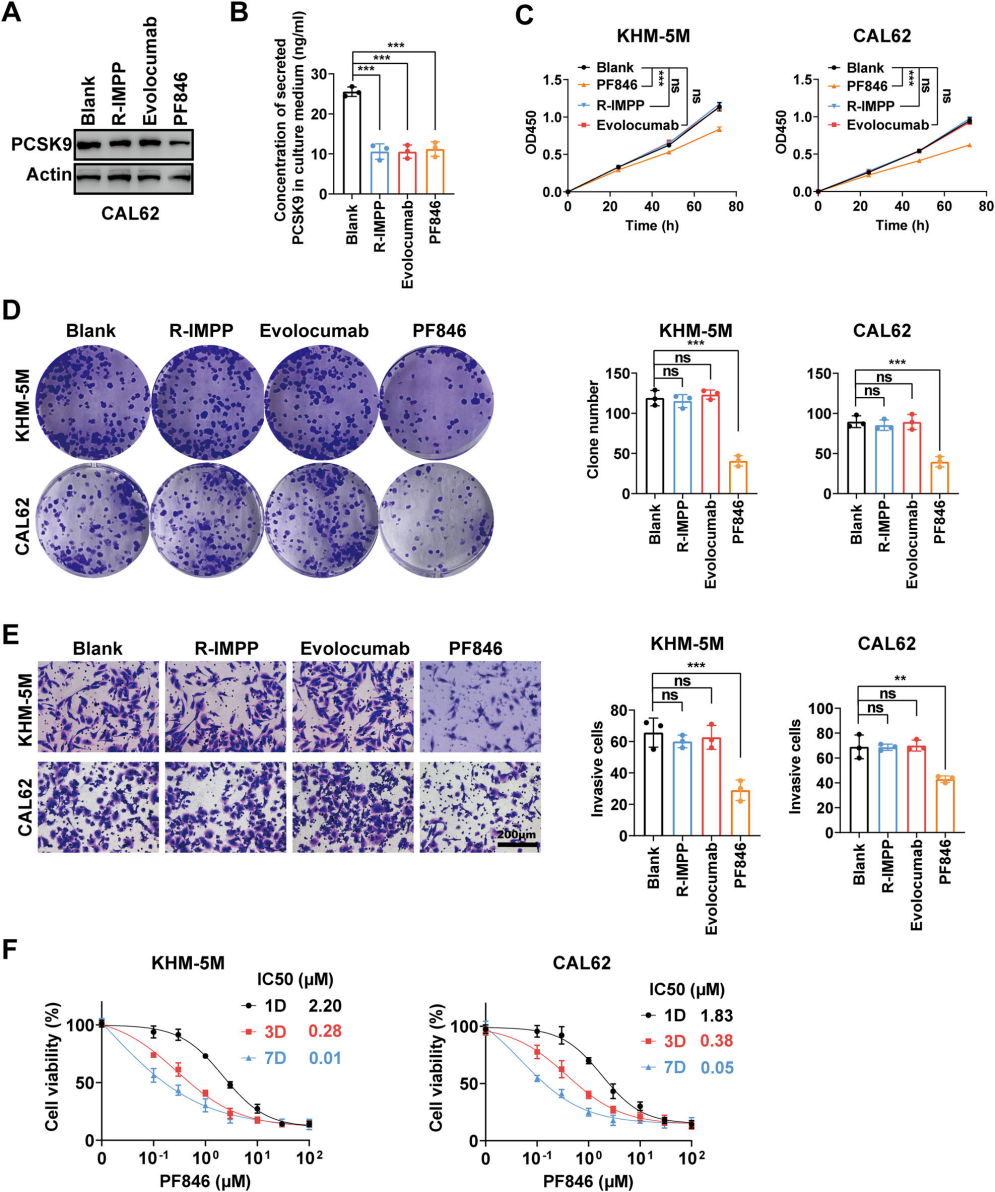
PCSK9/E-cadherin axis regulates malignant phenomenon of ATC through intracellular signaling. **A** Three PCSK9 inhibitors, R-IMPP, Evolocumab, and PF846, were selected to detect inhibition of PCSK9 protein levels. **B** The concentration of secreted PCSK9 in the culture medium was measured in the blank group and after treatment with three PCSK9 inhibitors, R-IMPP, Evolocumab, and PF846. **C** The proliferative abilities of KHM-5M and CAL62 cells detected by CCK8 assay and quantified after treatment with three PCSK9 inhibitors, R-IMPP, Evolocumab, and PF846. **D** The clone formation of KHM-5M and CAL62 cells assessed and quantified after treatment with three PCSK9 inhibitors, R-IMPP, Evolocumab, and PF846. **E** The invasive abilities of KHM-5M and CAL62 cells detected by Transwell assay and quantified after treatment with three PCSK9 inhibitors, R-IMPP, Evolocumab, and PF846. **F** IC50 of PF846 in KHM-5M and CAL62 cells detected after treatment with 1, 3, 7 days. Statistical analysis of the data from 2 groups was conducted with Student’s *t* test. Comparisons among multiple groups were conducted with one-way ANOVA followed by Fisher’s LSD test. (**p < 0.01, ***p < 0.001).

As PCSK9 is a secreted protein, we aimed to clarify whether it promotes ATC progression intracellularly or via autocrine mechanisms. To investigate this, we selected three PCSK9 inhibitors—R-IMPP, Evolocumab, and PF846—which target PCSK9 expression through different mechanisms. R-IMPP inhibits PCSK9 secretion, Evolocumab acts as a monoclonal antibody against PCSK9, and PF846 inhibits PCSK9 translation, with the first two primarily reducing extracellular PCSK9 levels. The results indicated that only PF846 significantly inhibited PCSK9 protein levels in CAL62 cells (Fig. 6A); in contrast, all three reduced extracellular PCSK9 levels, as detected by ELISA (Fig. 6B). Only PF846 significantly inhibited the proliferation and colony formation abilities of KHM-5M and CAL62 cells (Fig. 6C, D). Additionally, significant inhibition of the invasive ability of KHM-5M and CAL62 cells was observed only after treatment with PF846 (Fig. 6E). Further examination of the dose-response curve of PF846 over time in both cell lines revealed that after 7 days of treatment, the dose-response curve values for KHM-5M and CAL62 cells were 0.01 and 0.05 μM, respectively, indicating a promising anti-tumor effect of PF846 in ATC (Fig. 6F). Therefore, we concluded that the PCSK9/E-cadherin axis regulates ATC malignancy through intracellular signaling rather than an autocrine mechanism.

### PCSK9 inhibitor represses ATC progression in vivo

**Fig. 7:**
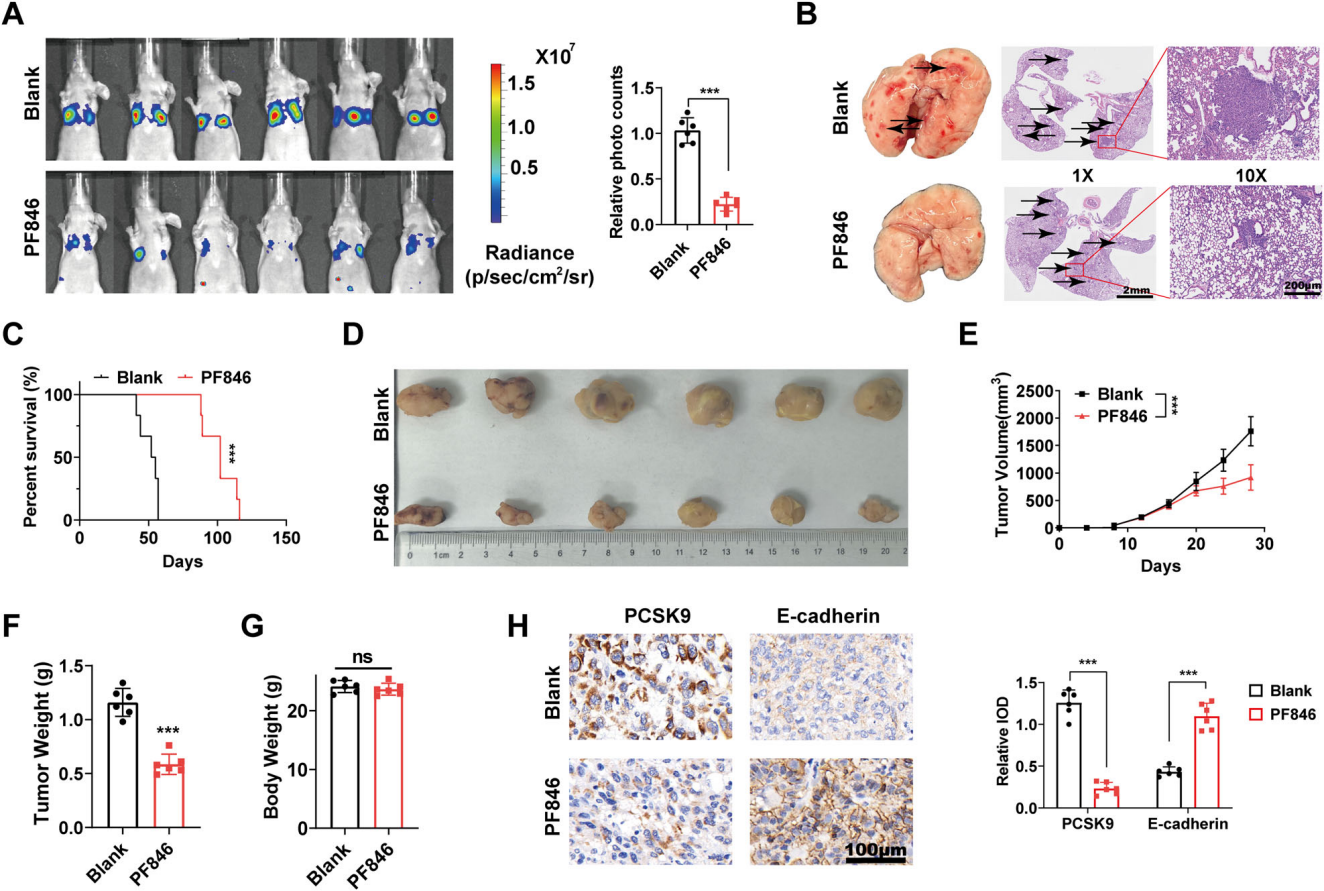
PCSK9 inhibitor represses ATC progression in vivo. **A** BALB/c nude mice injected with CAL62 cells (six mice for each group) via the tail vein were imaged at 40 days using an in vivo imaging system to evaluate overall metastasis and quantify differences between the Blank and PF846 (50 mg/kg, per 2 days, o.p.) treatment groups. **B** Representative images of H&E staining of lung metastasis loci. Scale bar = 200 μm. **C** Survival analysis of the BALB/c nude mice injected with cells via the tail vein between the Blank and PF846 (50 mg/kg, per 2 days, o.p.) treatment groups. **D** Image of subcutaneous tumor xenografts in Blank and PF846 treated groups. **E** The tumor growth curves for xenografts were graphed in the Blank and PF846 treated groups. **F** The tumor weights of xenografts were assessed. **G** The weight analysis of mice from blank and PF846 treated groups. **H** IHC staining and relative IOD of PCSK9 and E-cadherin in tumors from blank and PF846 treated groups. Scale bar = 100 μm. Statistical analysis of the data from 2 groups was conducted with Student’s *t* test. Comparisons among multiple groups were conducted with one-way ANOVA followed by Fisher’s LSD test. (**p < 0.01, ***p < 0.001).

Based on these in vitro findings, we investigated the in vivo efficacy of PF846. A metastatic animal model was established by tail vein injection to assess ATC cell metastasis. As depicted in Fig. 7A, B, using live imaging systems for small animals and H&E staining, we observed a significant inhibition of metastatic foci formation in the lungs of mice treated with PF846. Meanwhile, the expression level of PCSK9 in lung metastatic tumors was significantly suppressed in the PF846 treatment group (Fig. S2D). Additionally, the overall survival of mice receiving PF846 treatment was notably prolonged compared with that of the control group (Fig. 7C). As illustrated in Fig. 7D–F, PF846 markedly suppressed tumor growth in xenograft mouse models, with no observable alterations in the body weight of the treated mice (Fig. 7G). Furthermore, IHC staining revealed reduced PCSK9 expression in tumors from PF846-treated mice compared with controls; in contrast, the expression of E-cadherin exhibited an opposite trend (Fig. 7H).

## Discussion

ATC, though rare, is highly lethal and poses a major challenge in managing patients with TC [25, 26]. We identified the oncogenic role of PCSK9 in TC, particularly noting its significant overexpression in ATC tissues, which has important prognostic implications for patient survival. Validation using the TCGA database further confirmed the correlation between high PCSK9 expression and TC invasion and lymph node metastasis, consistent with the expression patterns observed in patients from FUSCC. ATC, characterized by rapid progression and invasiveness, exhibits elevated PCSK9 expression, promoting cholesterol metabolism as an energy source. Wong et al. previously reported that APC/KRAS-mutant colorectal cancer, the most aggressive subtype of colorectal cancer, also displays elevated PCSK9 expression, which supports cholesterol synthesis and energy utilization [16]. Our findings highlight the potential role of PCSK9-mediated cholesterol metabolism dysregulation in ATC, offering insights into future therapeutic strategies.

However, the specific mechanisms by which PCSK9 regulates tumor proliferation and invasion remain underexplored. Research by Xinjian Liu et al. demonstrated that PCSK9 disrupts the recycling of MHC-I to the cell surface by physically associating with it, facilitating its lysosomal degradation. Inhibition of PCSK9, either genetically or through antibodies, enhances MHC-I expression on tumor cells, suggesting that PCSK9 may influence tumor progression and therapeutic response via immune regulation. Our study uniquely shows that PCSK9 promotes tumor progression by directly regulating E-cadherin expression, enriching current research and elucidating its broader role in tumor biology.

E-cadherin, also known as epithelial cadherin, is a cell adhesion protein encoded by CDH1 that maintains cell-cell adhesion and tissue structure [27]. E-cadherin’s role in tumorigenesis and progression is well-documented, with its expression closely tied to cancer occurrence and prognosis [22, 28]. Our findings demonstrate that PCSK9 promotes ATC malignancy by facilitating E-cadherin degradation, primarily through ubiquitination and the lysosomal degradation pathway. In hepatocellular carcinoma, Lu et al. showed that basolateral CD147 induces hepatocyte polarity loss via E-cadherin ubiquitination and degradation [29]. Similarly, Zhang et al. found that TMEM139 interacts with E-cadherin in NSCLC, preventing its lysosomal degradation and inhibiting EMT [30]. Our work underscores the critical role of the PCSK9/E-cadherin axis in ATC progression, contributing to a deeper understanding of ATC pathogenesis.

The lysosomal degradation pathway is essential for maintaining protein homeostasis and degrading cellular waste [31, 32]. This process involves encapsulating proteins in endosomes via endocytosis, which then fuse with lysosomes, where hydrolytic enzymes degrade the contents [33, 34]. Although E-cadherin mRNA levels remained unchanged following PCSK9 overexpression, further investigation using the proteasome inhibitor MG132 and the lysosome inhibitor CQ revealed that inhibiting lysosomal activity significantly reverses E-cadherin degradation. These findings support the lysosomal pathway as a critical mechanism by which PCSK9 exerts its effects, aligning with previous studies linking PCSK9 to lysosomal regulation of MHC-I and PD-L1 expression, thereby promoting resistance to tumor immunotherapy [17, 35].

Dedifferentiation, a key biological process in tumor development, plays a central role in ATC genesis [36]. Many patients with ATC previously had PTC, and it is common to find coexisting ATC and PTC pathologies, underscoring dedifferentiation’s importance in ATC pathogenesis. Although the BRAF V600E mutation, prevalent in PTC, is less frequent in ATC, our preliminary research suggests that coexpression of BRAF V600E and TERT drives dedifferentiation and TC progression [37]. The marked upregulation of PCSK9 in ATC compared to PTC in this study suggests its involvement in dedifferentiation, highlighting its pivotal role in ATC pathogenesis.

As one of the most studied tumor suppressor genes, p53 has garnered considerable attention in cancer biology. Loss of p53 function is a hallmark molecular alteration distinguishing ATC from DTC. Current research estimates p53 mutation rates in ATC range from 50 to 80% [38, 39]. Missense mutations, which occur at hotspot sites such as R175, G245, R248, R249, R273, R282, H179, and Y220, account for approximately 30% of all p53 missense mutations [40]. A next-generation sequencing study of 341 genes from 117 patients with PDTC and ATC revealed a 73% mutation rate for p53 in ATC, with R248Q being the most common mutation. These findings align with our results [24], providing new insights into how p53 mutations inactivate its suppression of PCSK9, contributing to ATC progression.

We explored the therapeutic potential of PCSK9 inhibitors for ATC using three distinct agents. Although all significantly reduced extracellular PCSK9 levels, only PF846, which targets PCSK9 translation intracellular, effectively inhibited ATC proliferation and metastasis both in vitro and in vivo. Monoclonal antibodies Evolocumab and Alirocumab, approved for hyperlipidemia by the EMA and FDA, demonstrate PCSK9’s potential as a therapeutic target in cancer [41, 42]. Interest in small-molecule PCSK9 inhibitors has also grown, with PF-06446846 showing efficacy in reducing cholesterol levels and enhancing immunotherapy [43, 44]. Despite PF846’s specificity in this study, reports of off-target effects warrant further investigation to address potential clinical challenges [45]. Our findings support PCSK9 inhibition as a promising strategy for enhancing ATC treatment, extending its therapeutic value beyond immunotherapy.

## Conclusions

In summary, this study comprehensively evaluated the role of PCSK9 in ATC progression and metastasis. Furthermore, PCSK9 overexpression enhanced ATC cell proliferation and invasion in vitro by downregulating E-cadherin expression. We identified the endocytosis and lysosomal degradation pathways as the primary mechanisms through which PCSK9 regulates E-cadherin expression. The loss of the p53 function, which is highly prevalent in ATC, leads to the de-repression of *PCSK9* at the transcriptional level. Additionally, PF846, a promising intracellular PCSK9 inhibitor, demonstrated considerable therapeutic effects on ATC in vivo, inhibiting both tumor growth and metastasis.

## Supplementary information

Supplementary

Original WB images

## Data Availability

The datasets used and analyzed during the current study are available from the corresponding author on reasonable request.
