# Supplementary material for: PCSK9 promotes progression of anaplastic thyroid cancer through E-cadherin endocytosis
Source: Cell Death Dis. 2025 May 6;16(1):362. doi: 10.1038/s41419-025-07690-1 (PMC12056021; doi:10.1038/s41419-025-07690-1)
Supplement: Supplementary file 1 — Supplementary [file 41419_2025_7690_MOESM1_ESM.docx]

Supplementary data for

**PCSK9 promotes progression of anaplastic thyroid cancer through E-cadherin endocytosis**

**File list:**

1. Table S1

2. Table S2

3. Figure S1

4. Figure S2

1. Table S1. Primers used for qRT-PCR

| Gene | Forward (5'-3') | Reverse (5'-3') |
| --- | --- | --- |
| β-actin | TGACGTGGACATCCGCAAAG | CTGGAAGGTGGACAGCGAGG |
| PCSK9 | ATGGTCACCGACTTCGAGAAT | GTGCCATGACTGTCACACTTG |
| E-Cad | CGAGAGCTACACGTTCACGG | GGGTGTCGAGGGAAAAATAGG |
| P53 | CAGCACATGACGGAGGTTGT | TCATCCAAATACTCCACACGC |

qRT-PCR, Quantitative Reverse Transcription Polymerase Chain Reaction.

2. Table S2 Clinical features of patients enrolled in IHC staining

| **Variables** | **PCSK9 expression** | | **χ²** | **P value** |
| --- | --- | --- | --- | --- |
|  | Low(n=38) | High (n=38) |  |  |
| Gender |  |  | 0.054 | 0.815 |
| Female | 15(39.5) | 16(42.1) |  |  |
| Male | 23(60.5) | 22(57.9) |  |  |
| Age |  |  | 0.057 | 0.811 |
| <55 | 13(34.2) | 14(36.8) |  |  |
| **≥55** | 25(65.8) | 24(63.2) |  |  |
| Multifocality |  |  | 6.397 | 0.011 |
| Yes | 32(84.2) | 22(57.9) |  |  |
| No | 6(15.8) | 16(42.1) |  |  |
| Bilaterality |  |  | 2.621 | 0.105 |
| Yes | 32(84.2) | 26(68.4) |  |  |
| No | 6(15.8) | 12(31.6) |  |  |
| T stage |  |  | 23.619 | ＜0.001 |
| T1-2 | 27 (71.1) | 6 (15.8) |  |  |
| T3-4 | 11 (28.9) | 32 (84.2) |  |  |
| LNM |  |  | 3.713 | 0.054 |
| N0 | 12(31.6) | 5(13.2) |  |  |
| N1 | 26(68.4) | 33(86.8) |  |  |
| Distant metastasis |  |  | 8.491 | 0.004 |
| M0 | 34(89.5) | 23(60.5) |  |  |
| M1 | 4(10.5) | 15(39.5) |  |  |

PCSK9, Proprotein Convertase Subtilisin/Kexin Type 9; IHC, Immunohistochemistry; LNM, lymph node metastasis; χ², chi-square value

Among the 76 patients, 54 were PTC patients and 22 were ATC patients. Based on the median PCSK9 protein expression level from the IHC experiment, patients were classified into high and low expression groups. A combined analysis was conducted to assess the correlation between clinicopathological factors and PCSK9 expression levels.

3. Figure S1


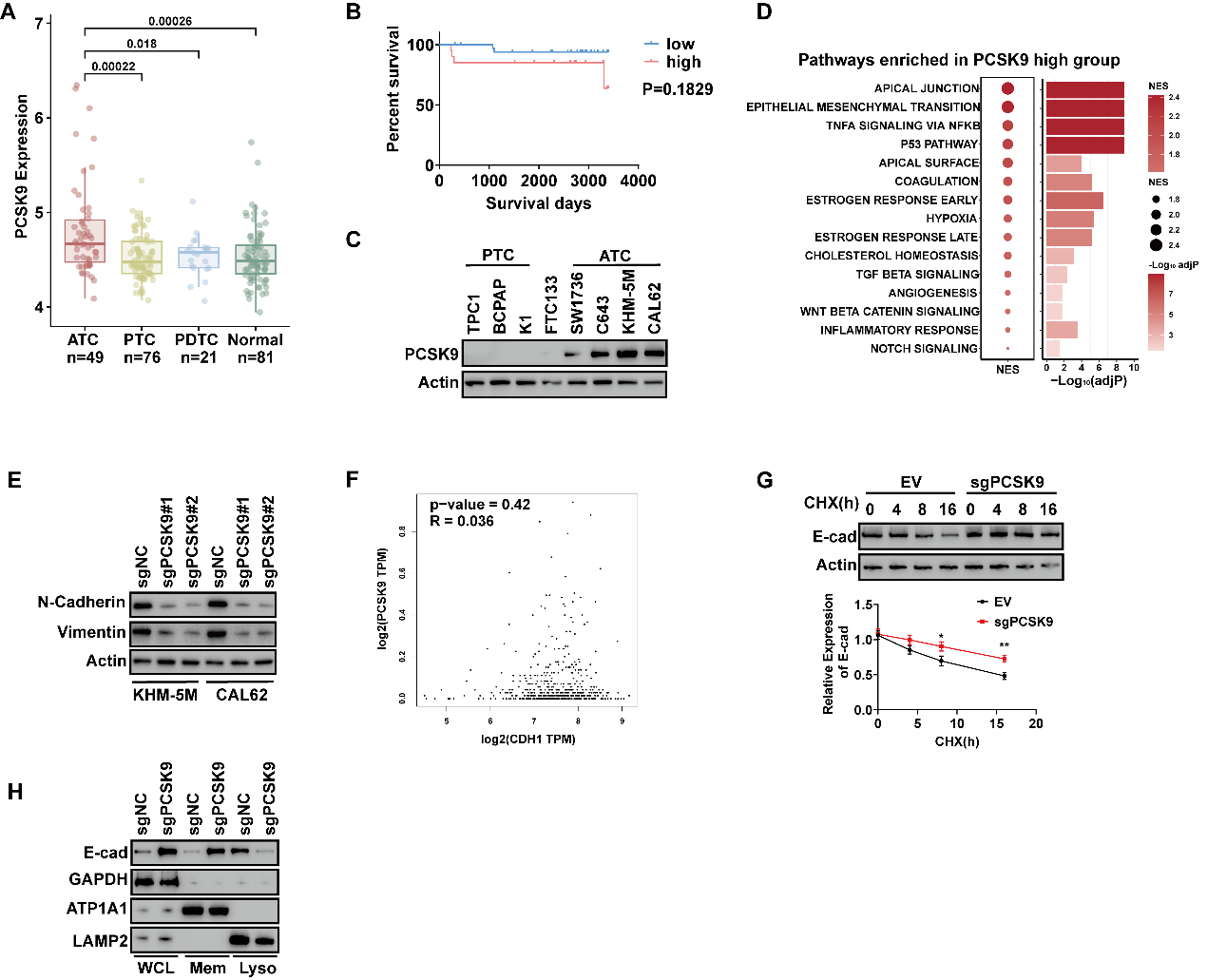


**Figure S1**

A. PCSK9 was significantly upregulated in ATC compared to PTC, PDTC, and normal thyroid tissues in the GEO database of 227 cases of thyroid cancer.

B. The association between PCSK9 and overall survival of 54 cases of PTC patients in FUSCC (Kaplan-Meier analysis).

C. The expression of PCSK9 across various thyroid cancer cell lines.

D. Gene Set Enrichment Analysis (GESA) showed that elevated PCSK9 expression was linked to various tumor proliferation and development pathways.

E. The knockout of PCSK9 inhibited the expression of N-cadherin and Vimentin, suggesting PCSK9 induced EMT process

F. PCSK9 and E-cadherin mRNA showed no significant correlation in thyroid cancer samples of the TCGA database.

G. Knock out of PCSK9 increased the protein stability of E-cadherin. Protein synthesis inhibitor Cycloheximide (CHX, 50 μg/mL).

H. Protein levels of E-cadherin, ATP1A1 and LAMP2 in WCL, Mem and Lyso of EV and PCSK9-knock out CAL62 cells.

Statistical analysis of the data from 2 groups was conducted with Student’s t test. **p < 0.01. PCSK, Proprotein Convertase Subtilisin/Kexin Type 9; EV, Empty vector; sgPCSK9, single guide PCSK9; CHX, cycloheximide; E-cad, E-cadherin; ATP1A1, ATPase Na+/K+ transporting subunit alpha 1; LAMP2, Lysosome-associated membrane glycoprotein 2; WCL, whole cell; Mem, membrane; Lyso, lysosome.

**Figure S2**


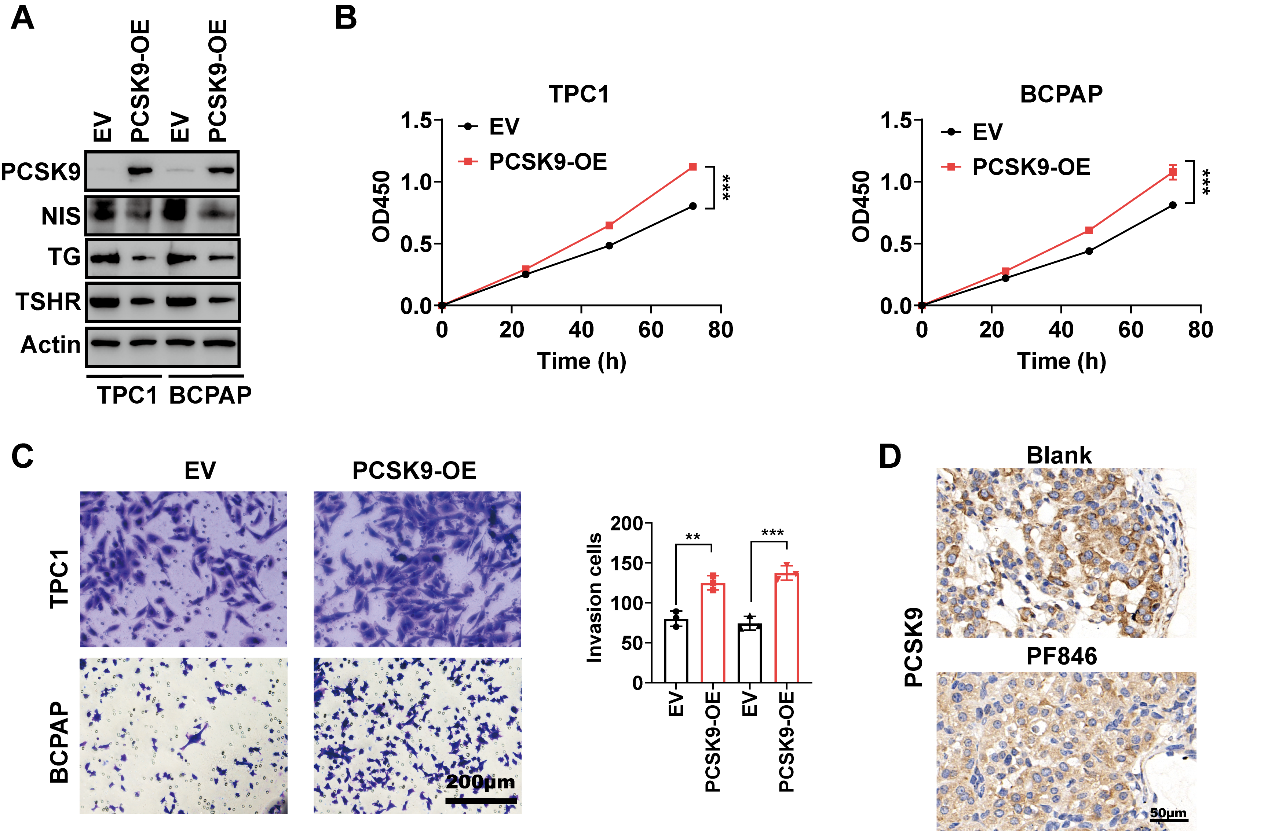


**Figure S2**

A. Overexpression of PCSK9 inhibits the protein levels of differentiation-related markers (NIS, TG, TSHR) in PTC cell lines (TPC1, BCPAP).

B. The proliferative abilities of EV and PCSK9-OE detected by CCK8 assay in TPC1 and BCPAP cells.

C. The invasive abilities of TPC1 and BCPAP cells detected by Transwell assay upon PCSK9 overexpression. Scale bar, 200 μm.

D. Representative images of PCSK9 IHC staining results in lung metastases from the blank group and the PF846 treatment group. Scale bar, 50 μm.

Statistical analysis of the data from 2 groups was conducted with Student’s t test. **p < 0.01, ***p < 0.001. NIS, Sodium/Iodide Symporter; TG, Thyroglobulin; TSHR, Thyroid-Stimulating Hormone Receptor.
