# Supplementary material for: PCSK9 promotes progression of anaplastic thyroid cancer through E-cadherin endocytosis
Source: Cell Death Dis. 2025 May 6;16(1):362. doi: 10.1038/s41419-025-07690-1 (PMC12056021; doi:10.1038/s41419-025-07690-1)

Figure2A

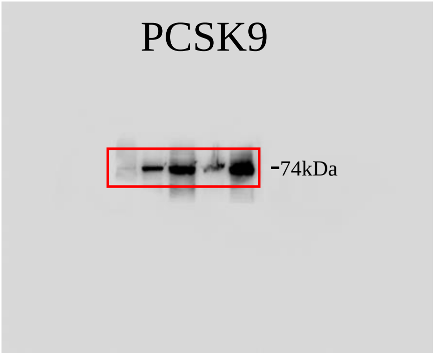

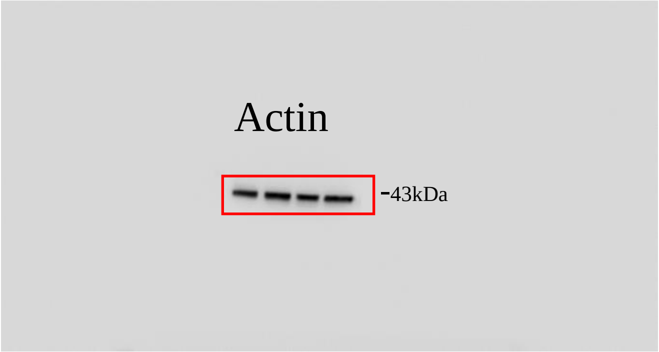

Figure2E

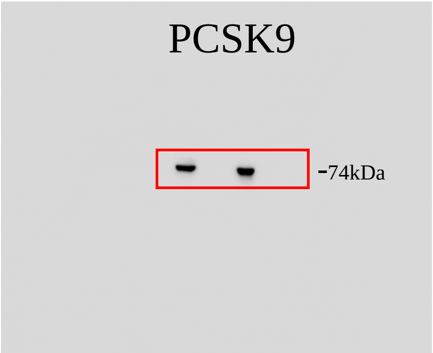

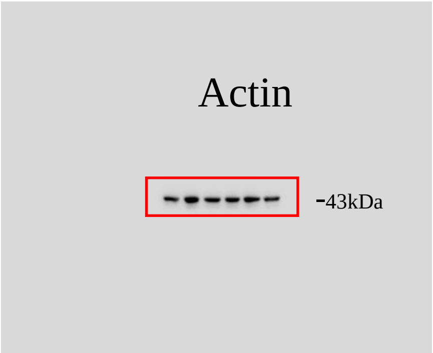

Figure3B

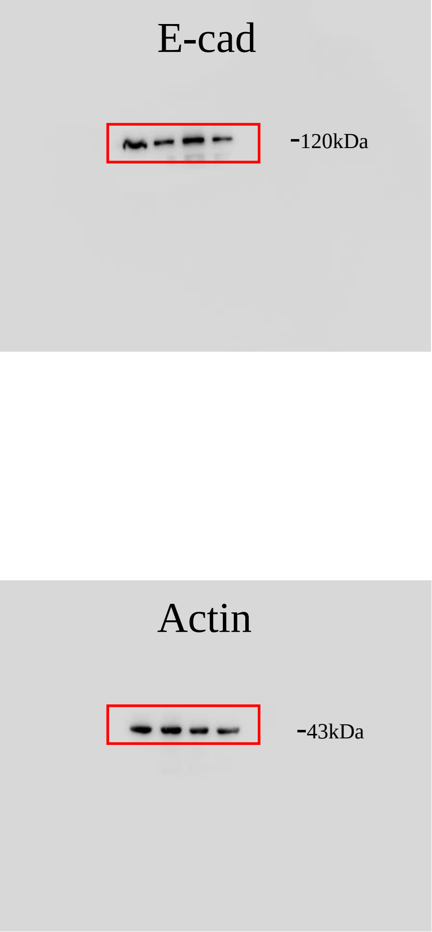

Figure3C

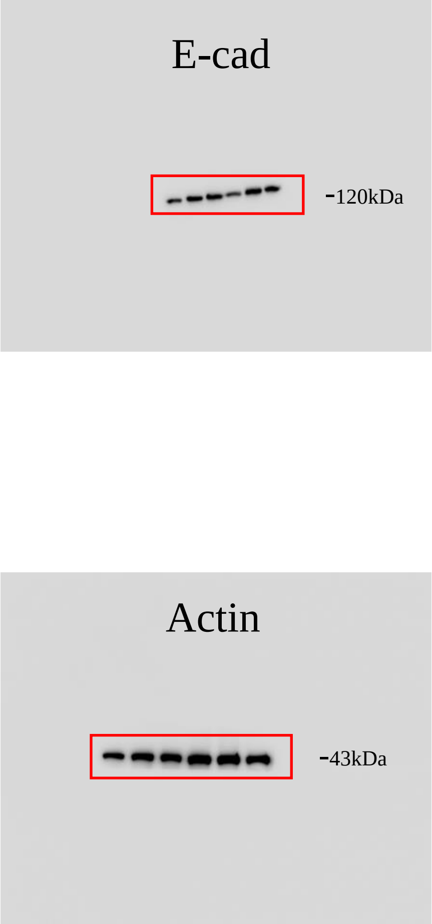

Figure3F

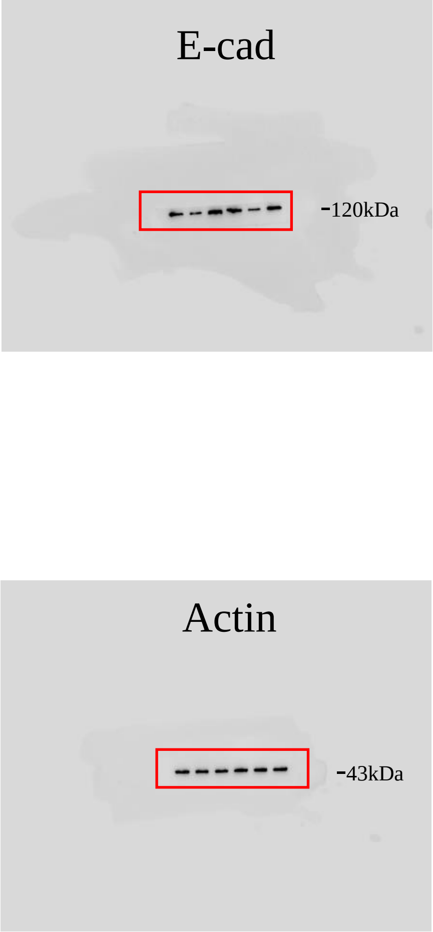

Figure4B

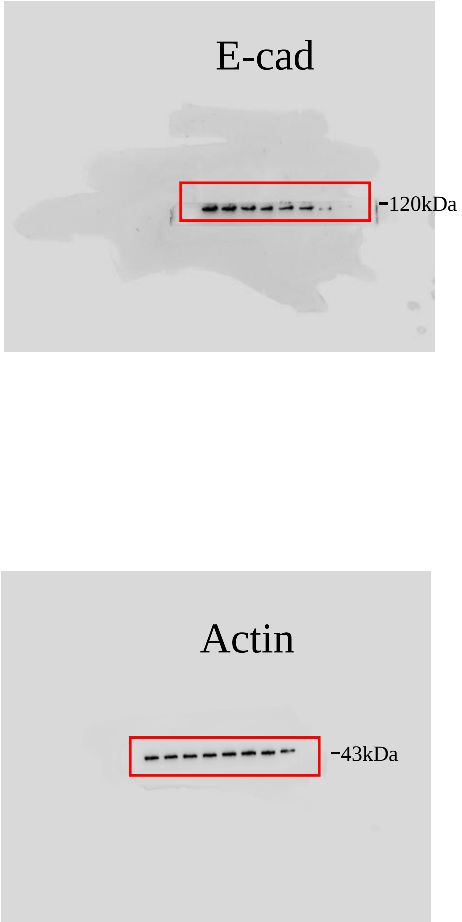

Figure4C

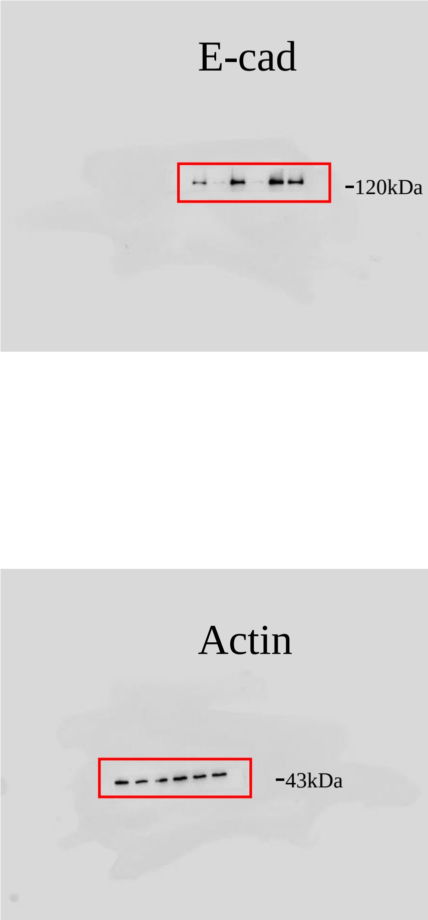

Figure4E

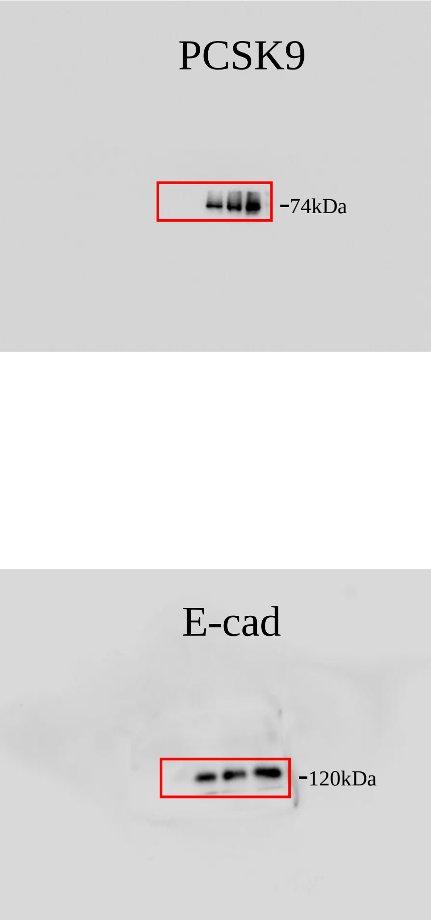

Figure4F

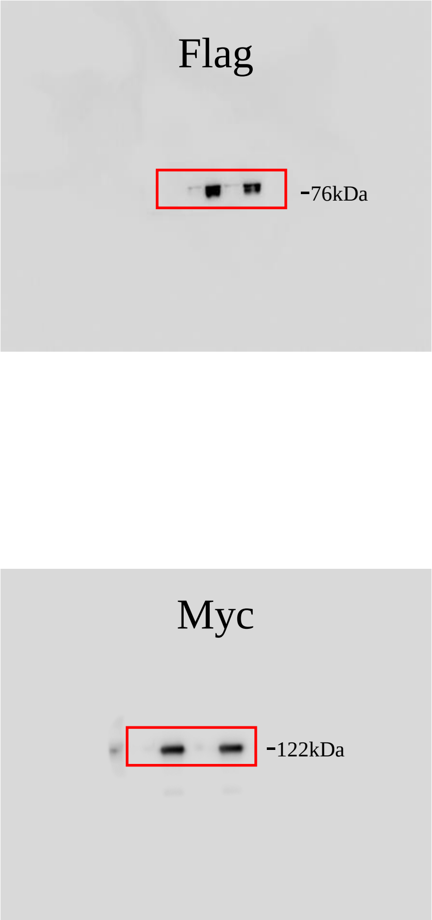

Figure4G

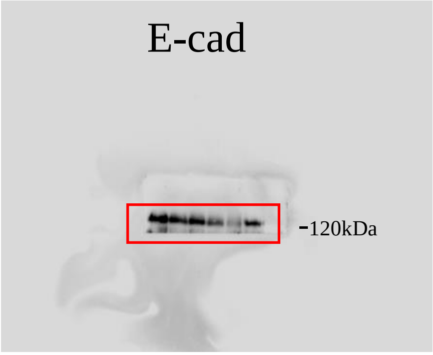

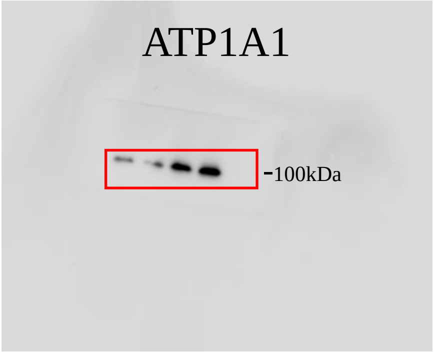

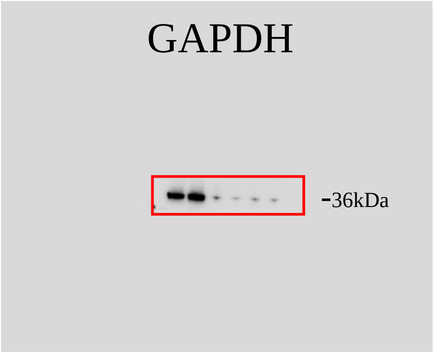

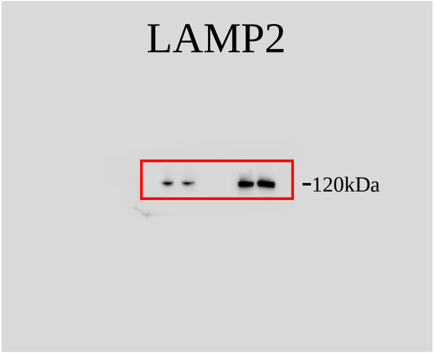

Figure5B

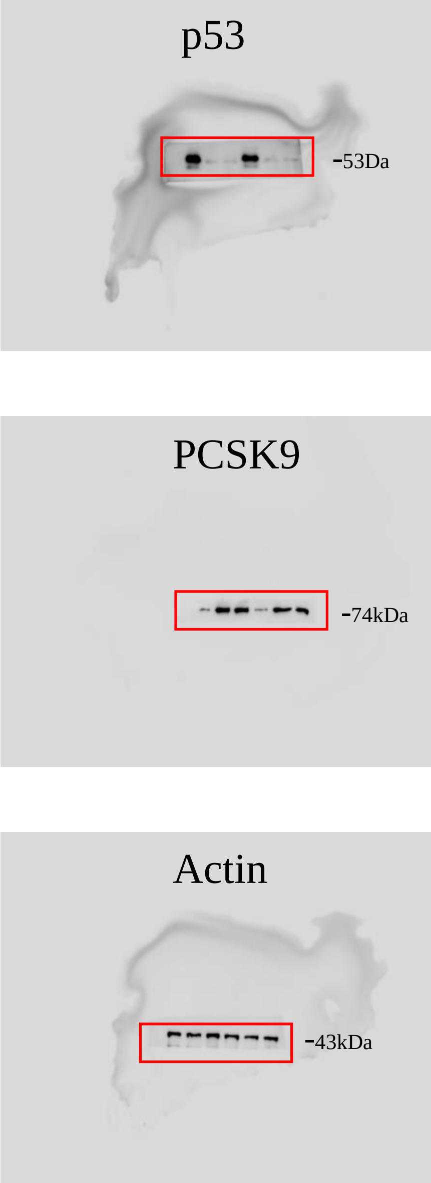

Figure5H

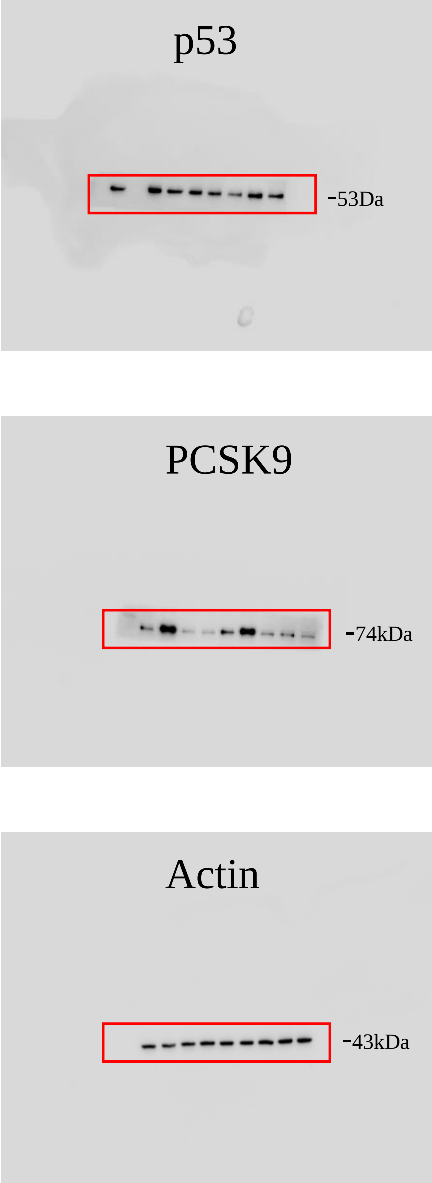

Figure6A

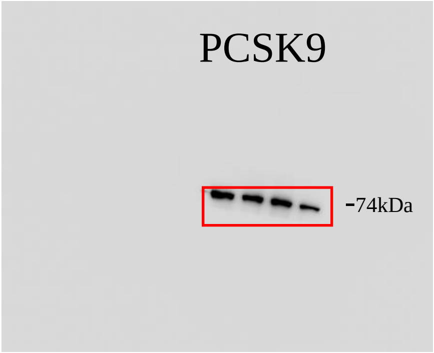

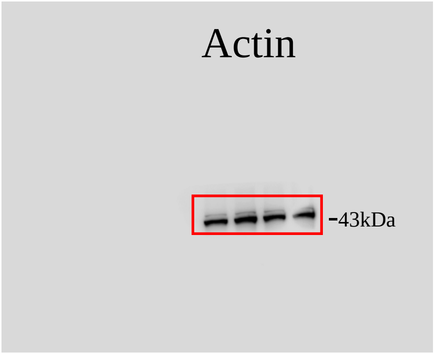

FigureS1C

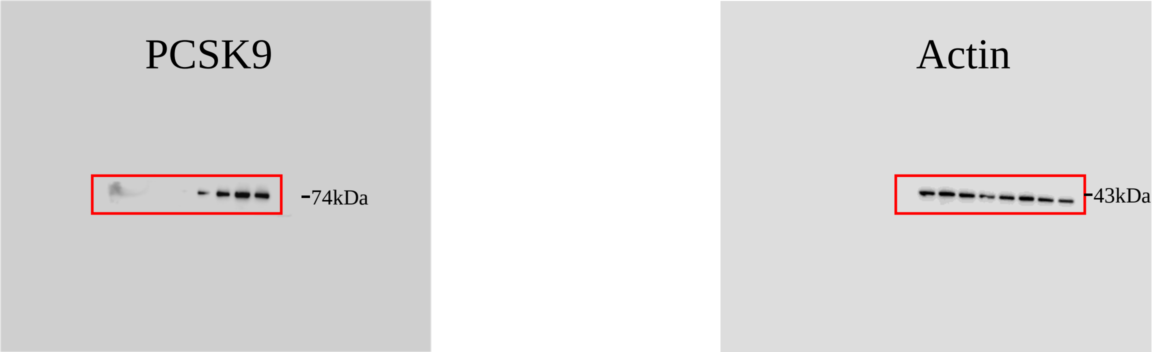

FigureS1E

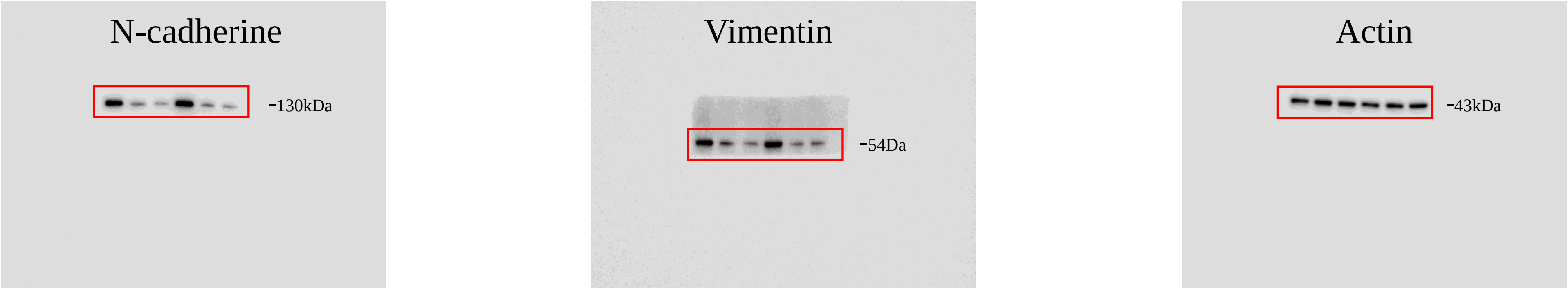

FigureS1G

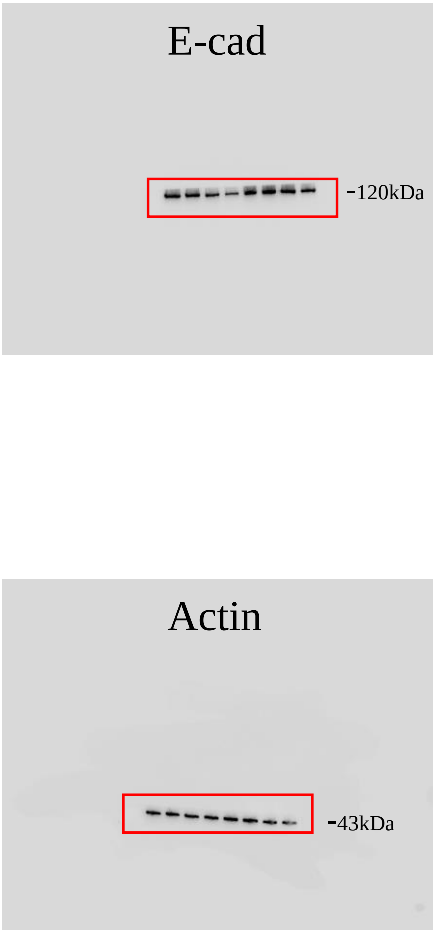

FigureS1H

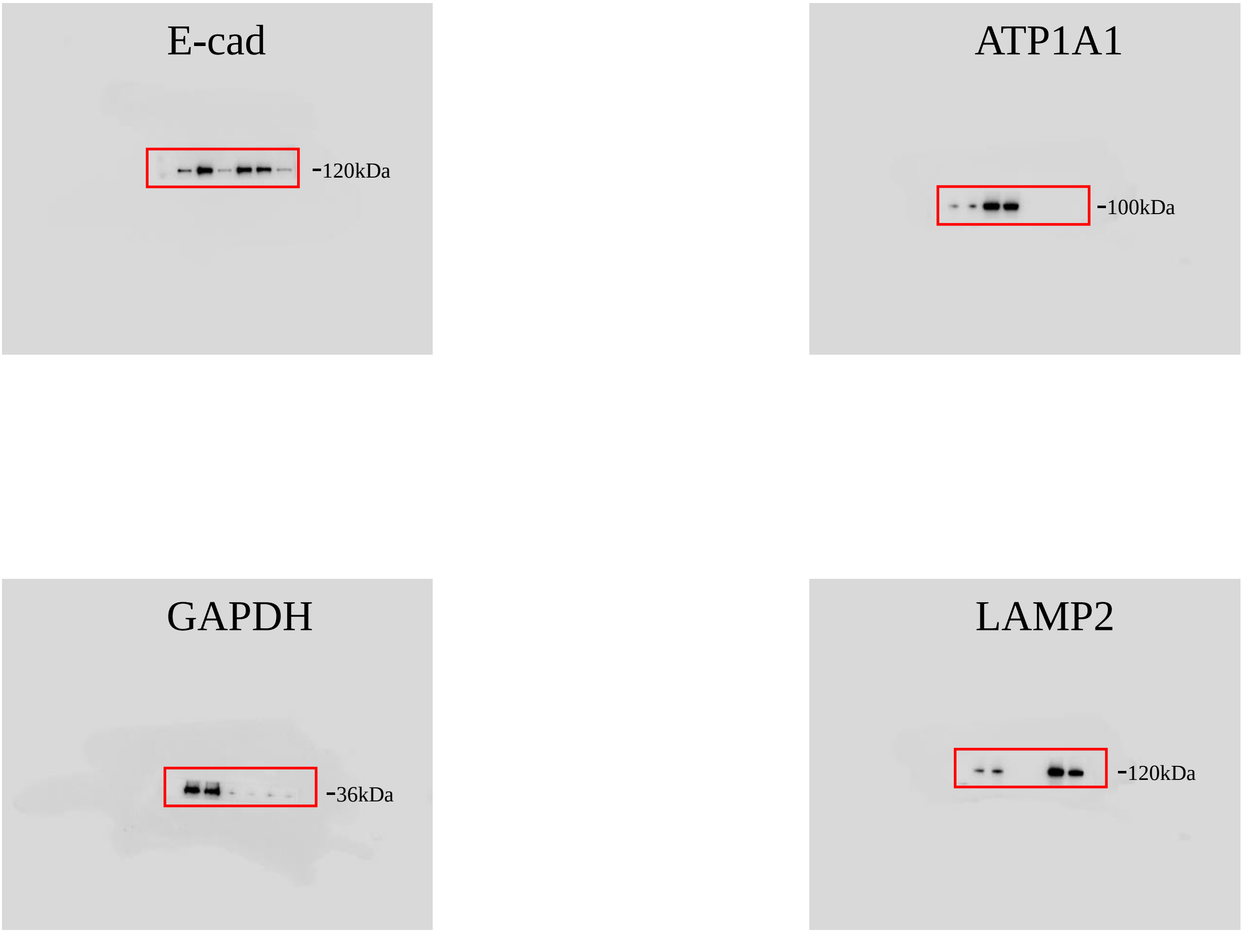

FigureS2A

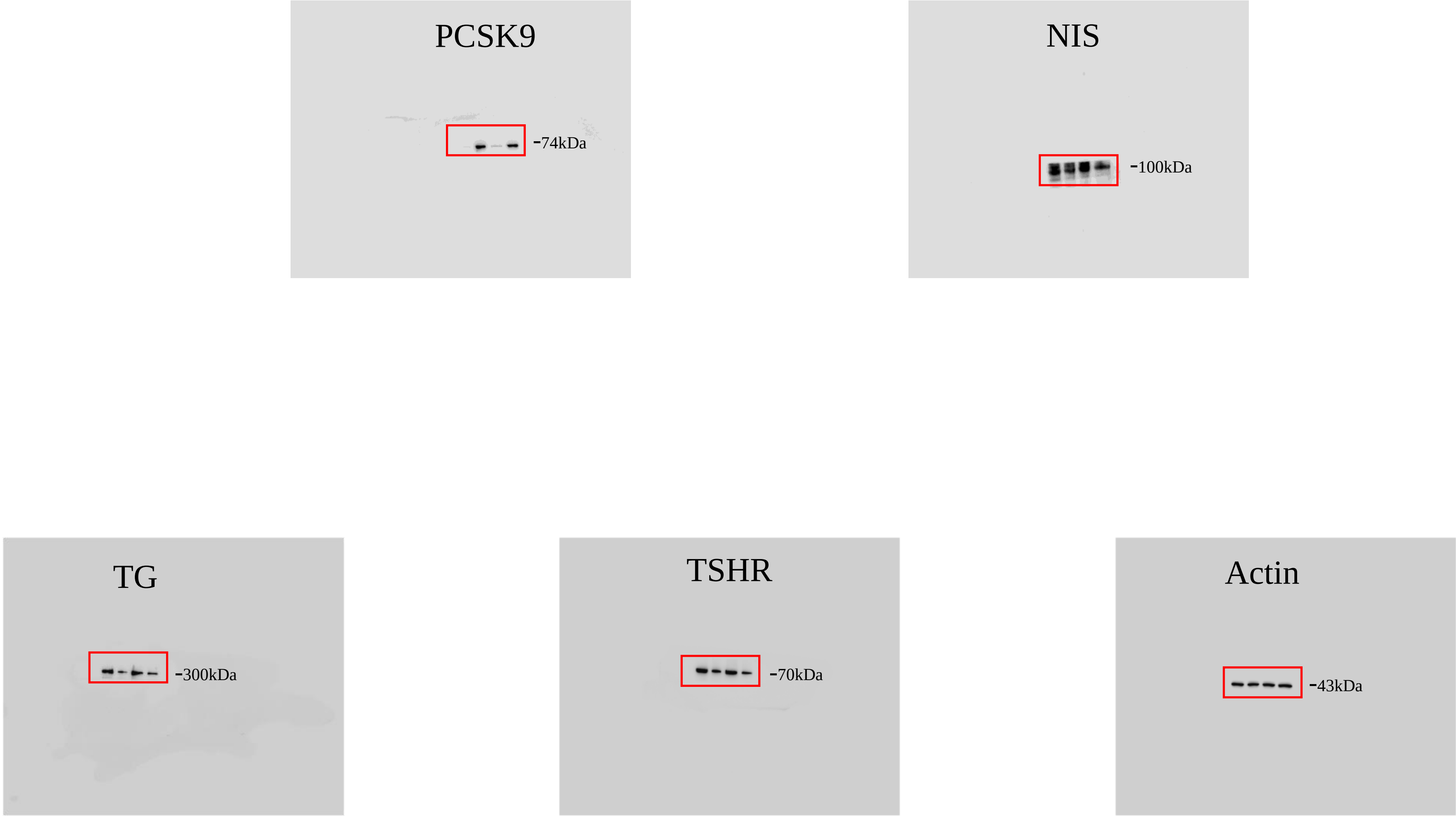

Supplement: Supplementary file 2 — Original WB images [file 41419_2025_7690_MOESM2_ESM.pdf]
